# Senescent Macrophages Promote Age‐Related Revascularization Impairment by Increasing Antiangiogenic VEGF‐A165B Expression

**DOI:** 10.1111/acel.70059

**Published:** 2025-04-17

**Authors:** Minghong Chen, Junyu Chen, Yu Liu, Xuerui Wang, Meilian Yao, Jing Chen, Jian Zhang, Qun Huang

**Affiliations:** ^1^ Department of Geriatric Medicine Xiangya Hospital, Central South University Changsha Hunan China; ^2^ Center of Coronary Circulation Xiangya Hospital, Central South University Changsha Hunan China; ^3^ National Clinical Research Center for Geriatric Disorders Xiangya Hospital, Central South University Changsha Hunan China; ^4^ Department of General and Vascular Surgery Xiangya Hospital, Central South University Changsha Hunan China; ^5^ Department of Cardiology Qilu Hospital of Shandong University Jinan Shandong China; ^6^ Department of Child Health Care Hunan Provincial Maternal and Child Health Care Hospital Changsha Hunan China

**Keywords:** angiogenesis, arteriogenesis, macrophages, peripheral arterial disease, revascularization, vascular endothelial growth factor A

## Abstract

Peripheral arterial disease is a common vascular disease in the elderly. Therapeutic revascularization, including angiogenic and arteriogenic therapy, is a promising treatment approach for peripheral arterial disease. However, the progress of clinical trials is not ideal, possibly due to insufficiency of preclinical models, such as not taking into account the effect of aging on vascular regeneration. Macrophages are crucial in angiogenesis and arteriogenesis. The aging microenvironment typically makes recruited monocytes and macrophages more susceptible to senescence. However, the feature of macrophages in ischemic hindlimb muscle of old individuals and their underlying role remains unclear. In this study, we reveal that macrophages of ischemic skeletal muscle in old mice are more senescent and proinflammatory. By transplanting macrophages into mice following hindlimb ischemia, we find senescent macrophages inhibit revascularization. Mechanistically, these senescent macrophages induce endothelial dysfunction via increasing vascular endothelial growth factor A‐165B (VEGF‐A165B) expression and secretion, and eventually impair revascularization. Notably, plasma VEGF‐A165B levels are elevated in old patients with PAD and positively associated with a lower ankle brachial index (ABI). Our study suggests that targeting the senescent macrophages presents an avenue to improve age‐related revascularization damage.

## Graphical abstract

The underlying mechanisms of age‐related revascularization impairment are not clear. Our research firstly identified that macrophages were more senescent in the ischemic skeletal muscle of old mice and senescent macrophages could inhibit revascularization. Mechanistically, these senescent macrophages induce endothelial dysfunction via increasing VEGF‐A165B expression and secretion, and eventually impair revascularization. Notably, plasma VEGF‐A165B levels are elevated in old patients with PAD and positively associated with a lower ankle brachial index.

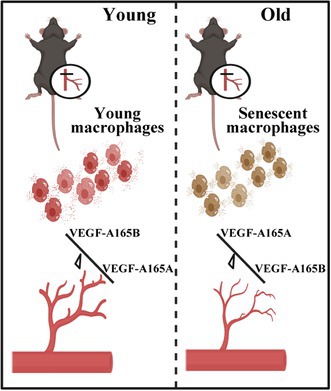

## 1 Introduction

Peripheral arterial disease (PAD) is a common vascular disease in the elderly, typically caused by atherosclerotic blockages that lead to acute and chronic ischemia, resulting in tissue damage (Golledge 2022). As global aging intensifies, the prevalence and incidence of PAD have markedly risen, affecting over 113 million middle‐aged and elderly individuals worldwide (Kim et al. 2023). Surgical revascularization (i.e., endovascular, surgical, or hybrid) is the current standard treatment for PAD but faces significant limitations due to associated comorbidities (Gornik et al. 2024). Therapeutic revascularization, including angiogenic and arteriogenic therapy, has emerged as a promising and highly anticipated treatment approach (Annex and Cooke 2021). However, current clinical trial progress is not optimistic, possibly due to deficiencies in preclinical models, such as the lack of consideration for risk factors like aging that impact angiogenesis/arteriogenesis (Cooke and Losordo 2015). The mechanisms by which aging impairs angiogenesis/arteriogenesis are complicated, necessitating further investigation to guide the development of new treatment strategies for PAD.

Macrophages play a pivotal role in the revascularization process following ischemic injury. Bone marrow‐derived monocyte‐macrophages are recruited early to ischemic and hypoxic sites (Arras et al. 1998; Heil et al. 2004), where they promote revascularization by secreting proangiogenic factors and regulating vascular and tissue repair through cell–cell communication (Takeda et al. 2011; Potente et al. 2011; Krishnasamy et al. 2017). Previous studies have shown that old mice exhibit impaired revascularization and more severe tissue damage following hindlimb ischemia (HLI) compared to young mice (Faber et al. 2011; Epstein et al. 2012). Aging reduces macrophage homing ability and alters the microenvironment, leading to delayed infiltration (Shavlakadze et al. 2010; Ahmadi et al. 2022); meanwhile, it causes dysfunction in monocyte‐macrophages (Moss et al. 2024; Duong et al. 2021), affecting impaired revascularization. Prior research indicated no significant difference in the number of monocyte‐macrophages in the hindlimb skeletal muscle of young and old mice post‐HLI (Faber et al. 2011), but their functionality was not evaluated. Whether the dysfunction of monocyte‐macrophages mediates the impaired revascularization and severe tissue damage in old mice post‐HLI, and the underlying mechanisms, requires further investigation.

Immunosenescence is particularly evident in the aging process and is closely associated with various age‐related diseases (Wang et al. 2024). Although current research on senescent macrophages is limited, some studies have shown that macrophages in old individuals exhibit characteristics of cellular senescence (Lin et al. 2018). The aging microenvironment leads to recruited monocyte‐macrophages being more prone to senescence (Becker et al. 2018; Blacher et al. 2022; Martini et al. 2019). In addition to exhibiting general features of cellular senescence, such as limited proliferation and the senescence‐associated secretory phenotype (SASP) (Dungan et al. 2022), senescent macrophages display specific characteristics, including impaired phagocytic function (Blacher et al. 2022), unbalanced polarization (Wang et al. 2015), dysregulated secretion (Li et al. 2021), and metabolic disorders (Minhas et al. 2019). Senescent macrophages contribute to the progression of age‐related diseases, such as neurodegenerative diseases (Natrajan et al. 2015; Rawji et al. 2020), diabetes (Bannon et al. 2013), and cardiovascular diseases (Childs et al. 2016). Macrophage senescence in old individuals may mediate age‐related revascularization impairment, necessitating further in‐depth research.

Vascular endothelial growth factor A (VEGF‐A) is a key molecule in revascularization following ischemic injury (Pérez‐Gutiérrez and Ferrara 2023). Due to the presence of two splicing isoforms, the role of VEGF‐A in revascularization is complex. Alternative splicing occurs at exon 8, where the proximal splice site produces the proangiogenic VEGF‐A165A isoform, while the distal splice site generates the antiangiogenic VEGF‐A165B isoform (Woolard et al. 2004). Vascular dysfunction in PAD patients is associated with elevated VEGF‐A165B and reduced VEGF‐A165A (Kikuchi et al. 2014). Previous studies have shown that the downregulation of VEGF‐A is related to age‐associated impairments in revascularization (Lähteenvuo and Rosenzweig 2012). In old mice, the expression of VEGF‐A and Neuropilin‐1, the key binding factor of VEGF‐A, are both decreased in the ischemic hindlimb muscles (Wagatsuma 2006). In human ischemic skeletal muscle, VEGF‐A expression also decreases with age (Ryan et al. 2006). Increasing VEGF‐A expression can improve age‐related revascularization impairment (Leosco et al. 2007). However, most studies have not addressed changes in VEGF‐A splice variants during aging. An important source of VEGF‐A is early‐infiltrating mononuclear macrophages (Willenborg et al. 2012). Macrophage‐secreted VEGF‐A165B has been shown to reduce revascularization in PAD (Kikuchi et al. 2014; Ganta et al. 2017, 2019). Macrophages of different phenotypes exhibit secretion heterogeneity (Li et al. 2021). This study aimed to investigate whether the imbalance in VEGF‐A splice variant proportions secreted by senescent macrophages is the key factor in age‐related revascularization impairment.

Here, we found that macrophages in the hindlimb skeletal muscle of old mice after HLI exhibited characteristics of cellular senescence. We demonstrated that senescent macrophages inhibited the process of revascularization in mice following HLI, potentially due to the increased expression of the VEGF‐A splice variant VEGF‐A165B in senescent macrophages. Plasma VEGF‐A165B protein levels were upregulated in the old patients with PAD, which is positively correlated with the severity of PAD.

## 2 Methods

### 2.1 Mononuclear Cell Isolation

We isolated mononuclear cells from the affected hindlimb skeletal muscles of male C57BL/6J mice 3 days after femoral artery ligation (FAL), with slight modifications to the protocol described by Krasniewski et al. (2022). Briefly, mice were euthanized and all hindlimb skeletal muscles were immediately dissected. The muscles were minced into a slurry on ice, then digested in a solution containing 1000 U/mL Collagenase II (17101015; Gibco, USA) at 37°C for 70 min, with gentle shaking every 5 min. Digestion was stopped by adding DMEM (C11995500bt; Gibco) containing 10% fetal bovine serum (FBS) (0500; ScienCell, USA). After centrifugation at 400 *g* for 5 min, the supernatant was collected. The pellet was resuspended in a digestion buffer containing 11 U/mL Dispase II (04942078001; Roche, USA) for a second round of digestion at 37°C for 30 min, with gentle shaking every 5 min. After stopping the digestion, the supernatant was collected by centrifugation at 400 *g* for 5 min and then passed through a 40 μm cell strainer (22‐363‐547; ThermoFisher, USA) to remove tissue debris.

### 2.2 Macrophage Sorting and Bulk RNA‐Sequencing

Mononuclear cells were isolated from the affected hindlimb skeletal muscles of 3‐month‐old and 24‐month‐old male C57BL/6J mice 3 days after FAL. After centrifugation at 500 *g* for 8 min, the cells were resuspended and counted, then incubated with anti‐F4/80 MicroBeads (130‐110‐443; Miltenyi Biotec, Germany) at 4°C for 30 min according to the manufacturer's instructions. F4/80‐positive macrophages were isolated using magnetic‐activated cell sorting (MACS) and total RNA was extracted by using TRIzol reagent (9109; TaKaRa, Japan). RNA‐sequencing (RNA‐seq) using DNBSEQ sequencing technology platforms was performed by BGI Genomics. Heatmap, Kyoto Encyclopedia of Genes and Genomes (KEGG), Gene Ontology (GO) analysis and Gene Set Enrichment Analysis (GSEA) were performed using the Dr. Tom Cloud Platform.

### 2.3 SPiDER‐βGal ^+^ Macrophage Flow Cytometric Analysis and Sorting

During muscle dissociation and digestion, SPiDER‐β‐gal reagent (SG03; Dojindo) was added to incubate for at least 1 h according to the manufacturer's protocol, with other dissociation steps performed as previously described. The prepared mononuclear cells were centrifuged and resuspended for counting. Based on the cell count, the cells were incubated with Aqua (1:100, 423101; Biolegend) at 4°C in the dark for 30 min. Then, anti‐CD16/CD32 (1:50, 156604; Biolegend) was used to block Fc receptors by incubating for 5 min. For macrophage‐related analysis, the cells were further stained with antibodies (APC‐CY7 antimouse CD45 [1:100, 557659; BD Pharmingen], PE antimouse CD11b [1:80, 101208; Biolegend], PE/Dazzle5944 anti‐mouse Ly‐6G [1:40, 127647; Biolegend], PerCP‐Cy5.5 antimouse Ly‐6C [1:100, 560525; Biolegend], APC antimouse F4/80 [1:80, 123116; Biolegend], PE‐Cy7 antimouse CD86 [1:80, 560582; BD Pharmingen], SPiDER‐βGal [1 μM, SG02; Dojindo]) at 4°C in the dark for 40 min. After permeabilization, the cells were stained with BV‐785 antimouse CD206 (1:40, 141729; Biolegend). The expression of specific cell markers was used to analyze the proportion of total macrophages and the ratios of senescent cells within CD86^+^ and CD206^+^ macrophages.

For sorting, young macrophages (CD45^+^/CD11b^+^/Ly‐6G^−^/Ly‐6C^−^/F4/80^+^/SPiDER^−^) and senescent macrophages (CD45^+^/CD11b^+^/Ly‐6G^−^/Ly‐6C^−^/F4/80^+^/SPiDER^+^) were isolated for injection into the hindlimb skeletal muscle of mice.

### 2.4 Hindlimb Ischemia and Laser Doppler Imaging

All animal experiments were conducted according to protocols approved by Xiangya Hospital of Central South University. All C57BL/6J mice were purchased from Hunan SJA Laboratory Animal Co. Ltd., and maintained on a 12‐h light/dark cycle from 6 a.m. to 6 p.m. The temperature in the animal housing facility was maintained at 21°C. FAL was performed on 3‐month‐old and 24‐month‐old male mice as previously described with some modifications (Limbourg et al. 2009). Briefly, mice were anesthetized with sodium pentobarbital (50 mg/kg, intraperitoneal injection), and the femoral artery was exposed via a skin incision. The femoral artery was doubly ligated at the proximal end near the groin and the distal end near the knee. The skin was then sutured, and the mice were monitored during recovery. Noninvasive laser Doppler perfusion imaging was used to assess the success of the surgery. Quantitative scoring of ischemia and movement was performed as described by Chalothorn et al. (2007).

### 2.5 Macrophage Transfer Experiments

On the third day following HLI, flow cytometry was employed to sort young macrophages (CD45^+^/CD11b^+^/Ly‐6G^−^/Ly‐6C^−^/F4/80^+^/SPiDER^−^) and senescent macrophages (CD45^+^/CD11b^+^/Ly‐6G^−^/Ly‐6C^−^/F4/80^+^/SPiDER^+^). Mononuclear cell suspensions from the affected limb muscles were centrifuged, resuspended, and counted, with 1 × 10^6^ cells resuspended in 100 μL of PBS without calcium and magnesium. These cells were then immediately injected into the affected hindlimb skeletal muscles of C57BL/6J mice 3 days post‐HLI.

### 2.6 Microfil and Bismuth/Gelatin Perfusions

After anesthesia, mice were intravenously injected with 400 units of heparin. Following thoracotomy, the thoracic aorta was exposed, and the mice were transected below the aortic arch to remove the upper body. A 6‐0 suture was placed below the aorta, and a cannula was inserted and secured with a knot. The aorta was perfused through the cannula with 10 mL of PBS containing adenosine (1 g/L), morphine (4 mg/L), and heparin (100 mg/mL), followed by 10 mL of 4% paraformaldehyde. Subsequently, 1.5 mL of catalyzed yellow Microfil (MV‐122; Flow Tech) or 50% bismuth/5% gelatin (Simons 2008) was injected. The aorta and vena cava were ligated to retain the perfusate in the vessels, and polymerization was allowed at room temperature for 30 min. The hindlimbs were then harvested and fixed in 4% paraformaldehyde. The following day, the tissue was dehydrated in a graded ethanol series (50%, 70%, and 100%). Finally, the samples were cleared in benzyl alcohol/benzyl benzoate (BABB) and imaged using a stereomicroscope (Leica) or microCT.

### 2.7 microCT Imaging

Samples were scanned using a desktop microCT (Hiscan XM Micro CT, Suzhou, China). The settings for the X‐Ray tube were 80 kV and 100 μA, with images captured at a resolution of 10 μm. A rotational step of 0.5° through a 360° angular range with 50 ms exposure per step was used. The image reconstruction was performed using Hiscan Reconstruct software from Suzhou, China, followed by analysis with Hiscan Analyzer software (Suzhou, China).

### 2.8 Immunofluorescence Staining

After perfusing with microfil, the hindlimb skeletal muscle tissues were fixed overnight at 4°C in 4% paraformaldehyde, then dehydrated, embedded, and sectioned. After being baked at 37°C for 1 h, tissue sections were rehydrated with PBS and permeabilized with PBS containing 0.1% Triton X‐100 for 10 min. The sections were blocked in PBS containing 10% donkey serum, 3% BSA, and 0.3% Triton X‐100 for 1 h at room temperature. Then, the sections were incubated overnight at 4°C with anti‐CD31 (1:200, AF3628; RD systems), anti‐F4/80 (1:200, 30325S; CST), and anti‐KI67 (1:150, 14‐5698‐82; ThermoFisher). The sections were washed three times with PBS, followed by incubation with Alexa Fluor‐conjugated donkey secondary antibodies (1:500; ThermoFisher) and antiactin, α‐Smooth Muscle Cy3 antibody (1:200, c6198; Sigma) for 1 h. All tissue sections were mounted with a DAPI‐containing mounting medium (ab104139; abcam). Immunofluorescent images were captured using a laser scanning confocal microscope (Zeiss LSM900, Germany).

### 2.9 Immunohistochemistry

After perfusing with microfil, the hindlimb skeletal muscle tissues were fixed in 4% paraformaldehyde at room temperature for 24 h, followed by dehydration, paraffin embedding, and sectioning. After the sections were deparaffinized, rehydrated, and antigen repaired, they were permeabilized with PBS containing 0.1% Triton X‐100 for 10 min. The enhanced polymer detection system kit (PV‐9000; ZSGB‐Bio) was used according to the manufacturer's instructions, and the primary antibody phospho‐eNOS (Ser1177) (1:200, AF3247; Affinity) was applied. The sections were then developed using DAB substrate (ZLI‐9019; ZSGB‐Bio). Finally, hematoxylin was used for nuclear counterstaining. Using ImageJ, we quantified the positive signals on the luminal surface of blood vessels, including all vessels perfused with microfil for the statistics.

### 2.10 Histological Analysis

After perfusing with microfil, the gastrocnemius muscles from the ischemic limb were harvested 21 days post‐HLI for histological analysis. The gastrocnemius muscles were immersed in 4% paraformaldehyde at room temperature for 24 h, followed by dehydration, paraffin embedding, and sectioning. Hematoxylin and eosin (HE) staining and Masson's trichrome staining were performed. Necrotic areas were characterized by necrotic muscle cells, inflammatory cells, and stromal cells, and the areas were quantified using ImageJ.

### 2.11 Bone Marrow‐Derived Macrophages Extraction and Senescence Induction

As previously described, bone marrow‐derived macrophages (BMDMs) were obtained from the femurs and tibias of mice (Assouvie et al. 2018). Briefly, femurs and tibias from both legs of 6‐week‐old male C57BL/6J mice were isolated and flushed with RPMI 1640 (C11875500BT; Gibco, USA). Red blood cells were lysed using 1 × RBC lysis buffer (00‐4333‐57; eBiosciences, San Diego, CA, USA), and the cells were washed with RPMI 1640 containing 10% FBS. The cells were then resuspended in differentiation medium consisting of RPMI 1640 (Gibco) supplemented with 10% FBS, 20% L929 conditioned medium (CM), and 1% penicillin/streptomycin (P/S). The cells were cultured in an incubator at 37°C with 5% CO_2_. The medium was changed on Day 3. The cells were harvested on Day 5 and transferred to six‐well plates for an additional 2 days of culture (L929 CM: L929 cells were cultured in low‐glucose DMEM containing 10% FBS and 1% P/S) at 37°C and 5% CO_2_ for 7 days. The supernatant was collected, filtered, and stored at −20°C until use.

Mature BMDMs were stimulated with 50 μM hydrogen peroxide (H_2_O_2_) for 24 h to establish a drug‐induced senescent cell model. The supernatants (labeled as “Y‐CM” for young macrophage‐CM and “S‐CM” for senescent macrophage‐CM) were collected for treating endothelial cells (ECs). Cells were harvested for total RNA and protein extraction for subsequent experiments.

### 2.12 Senescence‐Associated Beta‐Galactosidase and Immunocytochemistry Staining of BMDMs

After establishing the drug‐induced cellular senescence model, cell senescence was assessed using the senescence‐associated beta‐galactosidase (SA‐β‐Gal) staining kit (C0602; Beyotime) following the manufacturer's instructions. Immunocytochemistry involved fixing cells with 4% PFA at room temperature for 10 min, permeabilizing with PBS containing 0.1% Triton X‐100 for 10 min, blocking with PBS containing 1% BSA and 0.3% Triton X‐100 for 10 min, and incubating overnight at 4°C with primary antibodies (anti‐γ‐H2AX [1:200, 9718S; CST], anti‐KI67 [1:150, 14‐5698‐82; ThermoFisher], anti‐F4/80 [1:200, 30325S; CST]). After washing cells three times with PBS, Alexa Fluor‐conjugated donkey secondary antibodies (1:500; ThermoFisher) were incubated at room temperature for 30 min. Following DAPI staining, cells were imaged using fluorescence microscopy (Leica, Germany).

### 2.13 Western Blot Analysis

Total protein was extracted using RIPA buffer (P0013B; Beyotime) supplemented with a protease and phosphatase inhibitor cocktail (B15002; Bimake). Protein concentration was determined using a BCA protein assay kit (P0009; Beyotime). Proteins were separated by SDS‐PAGE and transferred onto PVDF membranes. Membranes were blocked with TBST containing 5% nonfat milk at room temperature for 1 h, then incubated overnight at 4°C with primary antibody (anti‐LMNB1, 1:1000, ab16048; Abcam; P21, 1:1000, ZRB1141; Sigma‐Aldrich; P16, 1:1000, ZRB1437; Sigma‐Aldrich; MHCII, 1:1000, bs‐8481R; Bioss; LYVE, 1:1000, 28,321‐1‐AP; Proteintech; VEGF‐A165B, 1:1000, MAB3045; R&D Systems; β‐actin, 1:5000, sc‐47778; Santa Cruz Biotechnology), then washed with TBST three times, and then incubated with secondary antibody (1:8000, ab6721; abcam) at room temperature for 1 h. Bands were visualized using a gel documentation system (Bio‐Rad, USA) and quantified using ImageJ software.

### 2.14 RNA Analyses

Briefly, total RNA of BMDM was extracted by using TRIzol reagent and reverse transcribed into cDNA using PrimeScript RT reagent Kit with gDNA Eraser (RR047A; Takara). Real‐time quantitative PCR (qPCR) was performed using the TB Green *Premix Ex Taq* (RR420A; Takara) with primers listed in Table S1.

### 2.15 Isolation of Endothelial Cells

Primary ECs from skeletal muscle (mECs) were isolated from 8‐ to 10‐week‐old male C57BL/6J mice. The dissociation of mouse hindlimb skeletal muscles and preparation of single‐cell suspensions were performed as previously described. After centrifugation and washing, the heterogeneous cell population was purified using flow cytometry and puromycin selection as previously described with minor modifications (Zhang et al. 2020). CD31‐positive cells were used for further experiments. mECs were incubated in endothelial cell medium (ECM) complete medium (ECM basic with 5% FBS, 1% ECGS and 1%P/S/Y‐CM/S‐CM), CM from senescent BMDMs transfected with 20 μM NC siRNA or *Vegf‐a165b* siRNA (GENERAL BIOL, China); S‐CM + 50 ng/mL IgG (MAB002; R&D Systems) or S‐CM + 50 ng/mL VEGF‐A165B antibody (MAB3045; R&D Systems). The amount of VEGF‐165 antibody was based on the studies by Ganta et al. (2019).

### 2.16 Assessment of Nitric Oxide (NO) Produced

8‐ to 10‐week‐old male C57BL/6J mice were injected with young or senescent primary macrophages 3 days after HLI. On Day 7, ECs were extracted from the ischemic hindlimb skeletal muscles, and nitric oxide (NO) levels were immediately measured using a NO detection kit (S0021S; Beyotime) according to the manufacturer's instructions.

mECs cultured in ECM complete medium (Ctrl), Y‐CM, or S‐CM; CM from senescent BMDMs transfected with NC siRNA or *Vegf‐a165b* siRNA; S‐CM + IgG or S‐CM + VEGF‐A165B antibody were collected and labeled with DAF‐FM DA probe (S0019S; Beyotime). The nitric oxide (NO) levels produced by the ECs were then analyzed using flow cytometry.

### 2.17 Aortic Sprouting Assay

8‐ to 10‐week‐old male C57BL/6 mice were euthanized. The thoracic aorta was isolated, perfused with sterile PBS to remove blood, and then dissected under a microscope to remove any surrounding tissues. Approximately 0.5 mm thick sections were cut and embedded in reduced growth factor Matrigel (354230; Corning). The aortas were incubated at 37°C for 1 h, followed by culture in ECM complete medium, Y‐CM, or S‐CM; CM from senescent BMDMs transfected with NC siRNA or *Vegf‐a165b* siRNA; S‐CM + IgG or S‐CM + VEGF‐A165B antibody in a culture dish. After 7 days, images of aortic sprouting were captured under a microscope.

### 2.18 Transwell Assay

A 24‐well Transwell chamber ( 3422; Corning) was used for the Transwell assay. For each sample, the upper chamber of the Transwell was precoated with 50 μL of 0.25% BSA without P/S ECM medium, and 500 μL of ECM complete medium was added to the lower chamber. mECs that had been pretreated with ECM complete medium, Y‐CM, or S‐CM; CM from senescent BMDMs transfected with NC siRNA or *Vegf‐a165b* siRNA; S‐CM + IgG or S‐CM + VEGF‐A165B antibody were gently added to the upper chamber. The cells were incubated for 16 h. The cells in the lower chamber were fixed with 4% paraformaldehyde and stained with 0.01% crystal violet (C0121; Beyotime) at room temperature for 30 min. Migrated cells were observed and counted in five randomly selected fields under an inverted optical microscope (Leica).

### 2.19 Tube Formation Assay

The tube formation assay was performed in a 96‐well plate, with 70 μL of Matrigel added to each well and allowed to polymerize at 37°C for 15 min. mECs cultured in ECM complete medium, Y‐CM, or S‐CM; CM from senescent BMDMs transfected with NC siRNA or *Vegf‐a165b* siRNA; S‐CM + IgG or S‐CM + VEGF‐A165B antibody were seeded onto the 96‐well plate at a density of 1 × 10^4^ cells per well and maintained in the previous medium. After 8 h of incubation, images were captured and analyzed using ImageJ software to quantify the number of sprouts and tube length.

### 2.20 EdU Staining

EdU staining was conducted using the BeyoClick EdU Cell Proliferation Kit with Alexa Fluor 488 (C0071S; Beyotime) according to the manufacturer's protocol. Images were acquired with a fluorescence microscope (Leica).

### 2.21 Intra‐Bone Marrow Injection of Adeno‐Associated Virus

Recombinant adeno‐associated serotype 9 viruses (AAV‐9) with Lyz2 promoter for short hairpin RNA (shRNA) targeting *Vegf‐a165b* knockdown in macrophages (AAV‐*Lyz2*‐sh*Vegf‐a165b*) purchased from WZ Biosciences Company (Shandong, China). AAV‐*Lyz2*‐sh*Vegf‐a165b* were injected into the bone marrow of 24‐month‐old mice at the concentration of 5 × 10^10^ vg per leg. The control group was injected with scrambled shRNA control (AAV‐Scramble).

### 2.22 Enzyme‐Linked Immunosorbent Assay

Peripheral blood from mice was collected 3 days after FAL, and then centrifuged at 3000 rpm for 10 min to obtain plasma. The plasma was promptly aliquoted and stored at −80°C. Cell culture supernatants from young and senescent BMDMs were also collected, aliquoted, and stored at −80°C. VEGFA, VEGF‐A165A, and VEGF‐A165B levels in mouse or human peripheral blood plasma and mouse BMDM culture supernatants were measured using enzyme‐linked immunosorbent assay (ELISA) kits (mouse: VEGFA, AF2128‐A; VEGF‐A165A, AF0260‐MA; VEGF‐A165B, AF0260‐MA; human: VEGFA, AF9809‐A; VEGF‐A165A, AF0424‐HA; VEGF‐A165B, AF0431‐HA; Hunan AiFang, China) according to the manufacturer's instructions, and relative comparisons were calculated.

### 2.23 Human Clinical Samples

This study was approved by the Central South University Xiangya Hospital Biomedical Research Ethics Committee (No. 20207447). Human plasma samples were obtained from 25 patients with PAD. Characteristics of the patients were provided in Table S2. All clinical participants were confirmed in advance to be free of major organ diseases or exclusion criteria, such as pregnancy, dialysis, cancer, liver failure, and chemotherapy The study participants were contacted by vascular surgery staff and gave their informed consent.

### 2.24 Statistical Analysis

All statistical data were represented using bar or line graphs, with means ± SEM displayed. Significance was defined as *p* < 0.05. Unpaired *t*‐tests were used for comparisons between two groups. For experiments involving more than two groups, one‐way analysis of variance (ANOVA) with Bonferroni's multiple comparison test was employed. Laser Doppler imaging experiments were analyzed using two‐way ANOVA and Sidak's multiple comparison test. Spearman correlation analysis was adopted between the VEGF‐A165B levels and ankle brachial index (ABI). Statistical analyses were conducted using GraphPad Prism 9.0.

## 3 Results

### 3.1 Macrophages Are More Senescent in Ischemic Skeletal Muscle of Old Mice

**FIGURE 1:**
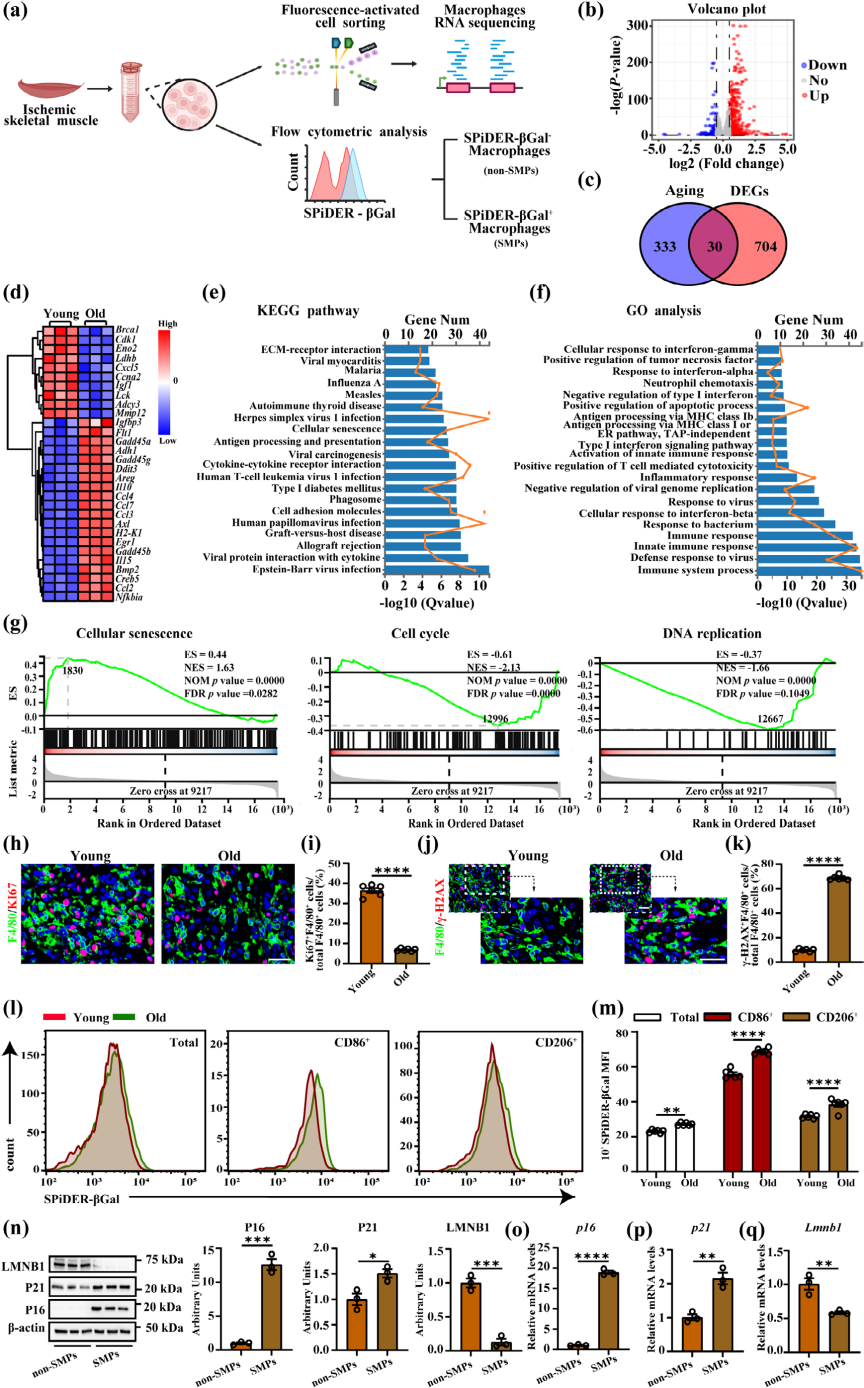
Aging alters the feature of macrophages in skeletal muscle. (a) Scheme illustrating experimental set‐up. (b) Volcano plot from RNA‐seq analysis showing differential gene expression of macrophages from hindlimb skeletal muscle of young (12 weeks) and old mice (24 months) 3 days after HLI (*n* = 3). (c) Venn diagram shows that 30 CS‐DEGs. (d) Heatmap of overlapping DEGs. (e) KEGG pathway enrichment analysis of DEGs. (f) GO analysis of DEGs. (g) GSEA of KEGG pathway. (h, i) Representative images of F4/80 (green), KI67 (red) and DAPI (blue) immunofluorescent images (h) and quantification (i) on gastrocnemius cross sections at 3 days after HLI (*n* = 6; scale bar = 25 μm). (j, k) Representative images of F4/80 (green), γ‐H2AX (red) and DAPI (blue) immunofluorescent images (j) and quantification (k) on gastrocnemius cross sections at 3 days after HLI (*n* = 6; scale bar = 25 μm). (l, m) Representative flow cytometry plots (l) and quantification (m) of the fluorescent intensity of SPiDER‐βGal in macrophages (*n* = 6). (n) Immunoblot images and quantification for P16, P21, or LMNB1 protein levels in non‐SMPs and SMPs isolated from hindlimb skeletal muscle of 24‐month‐old mice (*n* = 3). (o–q) Real‐time qPCR analysis of *p16 (o)*, *p21 (p)*, and *Lmnb1 (q)* in non‐SMPs and SMPs isolated from hindlimb skeletal muscle of 24‐month‐old mice (*n* = 3). Immunofluorescence staining, immunoblot, and Real‐time qPCR experiments were analyzed by Unpaired *t*‐tests. Fluorescent intensity of SPiDER‐βGal in macrophages was analyzed by one‐way ANOVA. Error bars represent SEM. *, **, ***, **** Denote *p* < 0.05, *p* < 0.01, *p* < 0.001, *p* < 0.0001, respectively.

To study the features of macrophages in ischemic skeletal muscle of old mice, we isolated macrophages in skeletal muscle from young (2 months) and old (24 months) mice 3 days after HLI and conducted RNA‐seq (Figure 1a). 734 differentially expressed genes (DEGs) were differentially regulated between them (Figure 1b). By cross‐analyzing the 734 genes with the CellAge database, we identified 30 overlapping cellular senescence‐DEGs (CS‐DEGs) (Figure 1c). The expression heatmap of these 30 CS‐DEGs could effectively distinguish between young and old mice (Figure 1d). DNA repair associated gene (*Brca1*) and cell cycle related genes (*Cdk1*, *Ccna2*) were significantly downregulated. SASP genes (*Il10*, *Ccl4*, *Ccl7*, *Ccl3*, *Il15*, *Ccl2*) and DNA damage‐related genes (*Ddit3*, *Gadd45a*, *Gadd45g*, *Gadd45b*) were significantly upregulated. Table S3 provided detailed information on these overlapping CS‐DEGs. KEGG pathway enrichment analysis revealed that DEGS were mostly enriched in cytokine–cytokine receptor interaction, cell adhesion molecules, cellular senescence (Figure 1e). GO analysis confirmed that DEGS were significantly enriched in immune system process, inflammatory response, and positive regulation of apoptotic process (Figure 1f). GSEA of KEGG pathway demonstrated that the pathway of cellular senescence was activated, but the pathway of cell cycle and DNA replication were suppressed in old mice (Figure 1g). Additional data indicated that macrophages in old mice exhibited less proliferation (Figure 1h,i) and severe DNA damage (Figure 1j,k).

The M1 (CD86^+^CD206^−^) or M2 (CD86^−^CD206^+^) were widely used to delineate the subtype of macrophages (Mantovani et al. 2002). However, recently, Krasniewski et al. (2022) showed using single‐cell RNA‐seq and flow cytometric analysis that CD86 and CD206 were coexpressed in most mice and human skeletal muscle macrophages. Their observations suggest that for skeletal muscle macrophages, M1 (CD86^+^CD206^−^) or M2 (CD86^−^CD206^+^) may not properly represent macrophage subtype (Cui et al. 2019; Krasniewski et al. 2022). Therefore, firstly, we analyzed the senescent condition of CD86 or CD206 single‐positive macrophages. To determine the nature of senescent macrophages in vivo, we further performed flow cytometry analysis to determine the expression of SA‐β‐gal activity in macrophages based on the fluorescent probe SPiDER‐βGal (Figure S1a). By contrast, in ischemic skeletal muscle of old mice, both total macrophages and CD86^+^ or CD206^+^ macrophages highly expressed the SPiDER‐βGal signal (Figure 1l,m). And we found that the old group had a higher total number of SPIDER‐β‐Gal^+^ macrophages and CD86^+^ SPIDER‐β‐Gal^+^ macrophages, while the number of CD206^+^ SPIDER‐β‐Gal^+^ macrophages was not significantly different between young and old groups (Figure S1b–j).

Meanwhile, we sorted nonsenescent macrophages (non‐SMPs) or senescent macrophages (SMPs) in ischemic skeletal muscle of old mice based on the SPiDER‐β‐gal. Analysis of western blot and qPCR showed that the increased expression of P16(Figure 1n,o), P21 (Figure 1n,p), and decreased LMNB1 (Figure 1n,q) in SMPs compared to non‐SMPs. Moreover, Krasniewski et al. thought that LYVE1 and MHCII are effective markers to delineate the subtype status of skeletal muscle macrophages, and the mRNA levels of *Mhcii* and *Lyve1* in skeletal muscle macrophages were significantly increased in old mice (Krasniewski et al. 2022). Then, we demonstrated that protein levels of MHCII and LYVE1 in SMPs were elevated (Figure S1k–m). Taken together, these data indicate that age promotes macrophages in ischemic skeletal muscle toward a senescent phenotype.

### 3.2 Senescent Macrophages Impair Revascularization in Young Mice Following Artery Occlusion

**FIGURE 2:**
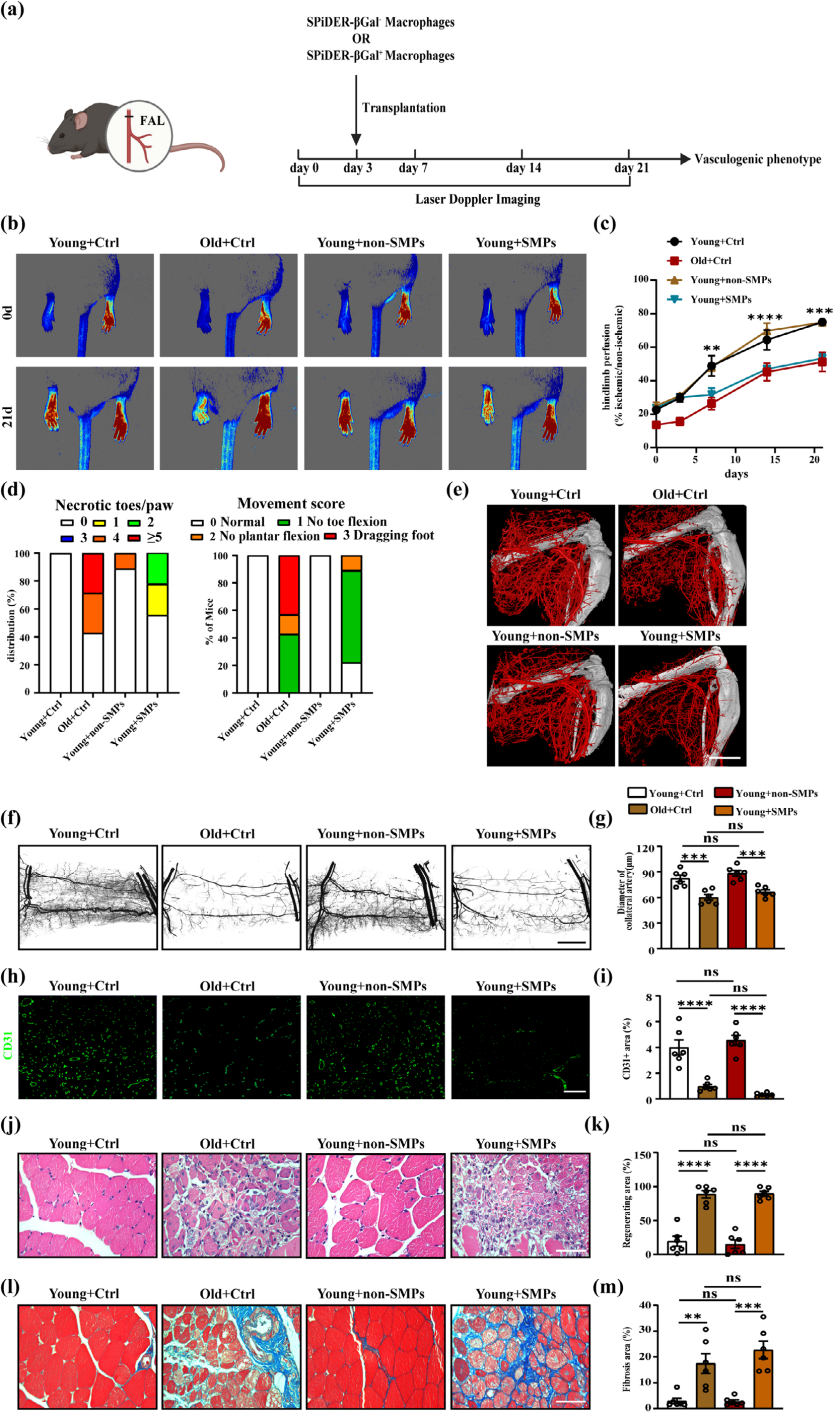
Senescent macrophages control ischemia‐induced revascularization. (a) Scheme showing macrophages transfer experiments. (b, c) Representative image (b) and quantification (c) of hindlimb blood perfusion of mice transferred with SMPs or non‐SMPs before and after HLI surgery (*n* = 6). (d) Distribution of necrosis toes per paw (*n* = 6) and movement score (*n* = 6) of mice transferred with SMPs or non‐SMPs at 21 days after HLI. (e) Representative microCT images of hindlimb vasculature of mice transferred with SMPs or non‐SMPs at 7 days after HLI (*n* = 6; scale bar = 4 mm). (f, g) Representative whole mount images (f) and quantification (g) of Microfil‐filled gracilis muscle vasculature from day 21 post‐HLI hindlimbs (*n* = 6; scale bar = 100 μm). (h, i) Representative CD31 immunofluorescent images (h) and quantification (i) on gastrocnemius cross sections of mice transferred with SMPs or non‐SMPs at 21 days after HLI (*n* = 6; scale bar = 100 μm). (j, k) Representative H&E staining images (j) and quantification (k) of the regenerating area on gastrocnemius cross sections of mice transferred with SMPs or non‐SMPs at 21 days after HLI (*n* = 6; scale bar = 75 μm). (l, m) Representative Masson staining images(l) and quantification (m) of fibrosis area on gastrocnemius cross sections of mice transferred with SMPs or non‐SMPs at 21 days after HLI (*n* = 6; scale bar = 75 μm). Laser Doppler imaging experiments were analyzed using two‐way ANOVA; other experiments were analyzed using one‐way ANOVA. Error bars represent SEM. *, **, ***, **** Denote *p* < 0.05, *p* < 0.01, *p* < 0.001, *p* < 0.0001, and ns, not significant respectively.

To investigate the potential role of senescent macrophages in revascularization, we performed adoptive transplantation experiments in young mice following HLI. We injected either PBS (Ctrl), non‐SMPs, or SMPs into the hindlimb of mice 3 days after HLI (Figure 2a). Laser Doppler imaging showed that hindlimb perfusion was not different between Young + Ctrl and Young + non‐SMPs. However, the transfer of SMPs impaired hindlimb perfusion (Figure 2b,c), which was similar with old mice. Moreover, the transfer of SMPs displayed a higher frequency of necrotic toes and a lower score of movement (Figure 2d). Concomitantly, microCT of the hindlimb displayed that SMPs reduced vascular density (Figure 2e). Whole‐mounted gracilis additionally showed that SMPs decreased the diameter of the preexisting collateral artery (Figure 2f,g), and immunofluorescence displayed that SMPs reduced the density of capillaries (Figure 2h,i). Importantly, the SMPs transfer led to striking acceleration of muscle regeneration (Figure 2j,k) and fibrosis (Figure 2l,m). Thus, senescent macrophages control age‐induced muscle revascularization dysfunction.

### 3.3 Senescent Macrophages Inhibit Proliferation and Activation of ECs In Vivo

Endothelial proliferation and activation play a crucial role during revascularization after injury. SMPs transfer inhibited arterial and capillary ECs proliferation, as indicated by fewer KI67^+^ α‐SMA^+^CD31^+^ cells (Figure S2a,b) and KI67^+^CD31^+^ cells in ischemic hindlimb muscle (Figure S2c,d). Phospho‐eNOS/NO (p‐eNOS/NO) is an important signaling pathway in developing revascularization. Transfer of SMPs decreased p‐eNOS expression in ischemic hindlimb muscle (Figure S2e,f). Next, we isolated ECs in ischemic hindlimb muscle to detect NO content, showing that NO content was lower in mice transferred with SMPs (Figure S2g). Therefore, these data suggest that senescent macrophages control revascularization, in part, in the hindlimb skeletal muscle of old mice via affecting the function of ECs.

### 3.4 Senescent Macrophages Induce ECs Dysfunction Ex Vivo

To further explore the impact of senescent macrophages on ECs communication, we developed an in vitro model using mouse primary BMDMs. After differentiation for 7 days (Figure S3a), BMDMs were subjected to immunofluorescent staining to confirm most cells express F4/80 (Figure S3b); the percent of F4/80^+^ cells in senescent BMDMs (SBMDMs) was relatively low (Figure S3c,d). Then, mature BMDMs were treated with H_2_O_2_ to induce a senescent model. Consistent with the senescent phenotype, SBMDMs exhibited significantly increased SA‐β‐Gal staining compared to control cells (YBMDMs) (Figure S3e,f). Analysis of western blot demonstrated decreased expression of the nuclear membrane marker LMNB1 and increased expression of the cyclin‐dependent kinase inhibitor P21 in SBMDMs compared to control cells (Figure S3g). The protein levels of MHCII and LYVE1 also increased in SBMDMs (Figure S3h).

There is also evidence shown that SBMDMs have less proliferation (Figure S3i,j) and severe DNA damage (Figure S3k,l). Analysis of qPCR additionally showed that the expression of chemokine components of the SASP, including *Ccl‐8*, *Ccl‐11*, *Cxcl12*, *Ccl13*, *Icam‐1*, *Il‐1α*, *Mmp‐10*, and *Tnf‐α*, and senescent markers, including *p15*, *p16*, *p19*, *p21*, *p27*, and *Cd38* were increased, and the expression of Sirts family, including *Sirt1*, *Sirt2*, *Sirt3*, *Sirt4*, *Sirt5*, *Sirt6*, and *Sirt7*, were decreased in SBMDMs (Figure S3m–p). Although some anti‐inflammatory marker (*Mgl1* and *Mgl2*) mRNA levels were increased, the expression of all proinflammatory markers were elevated (Figure S3q), suggesting that the SBMDMs were more proinflammatory, consistent with cellular senescence. What is more, the mRNA levels of *Mhcii* and *Lyve1* were increased in SBMDMs (Figure S3r,s).

**FIGURE 3:**
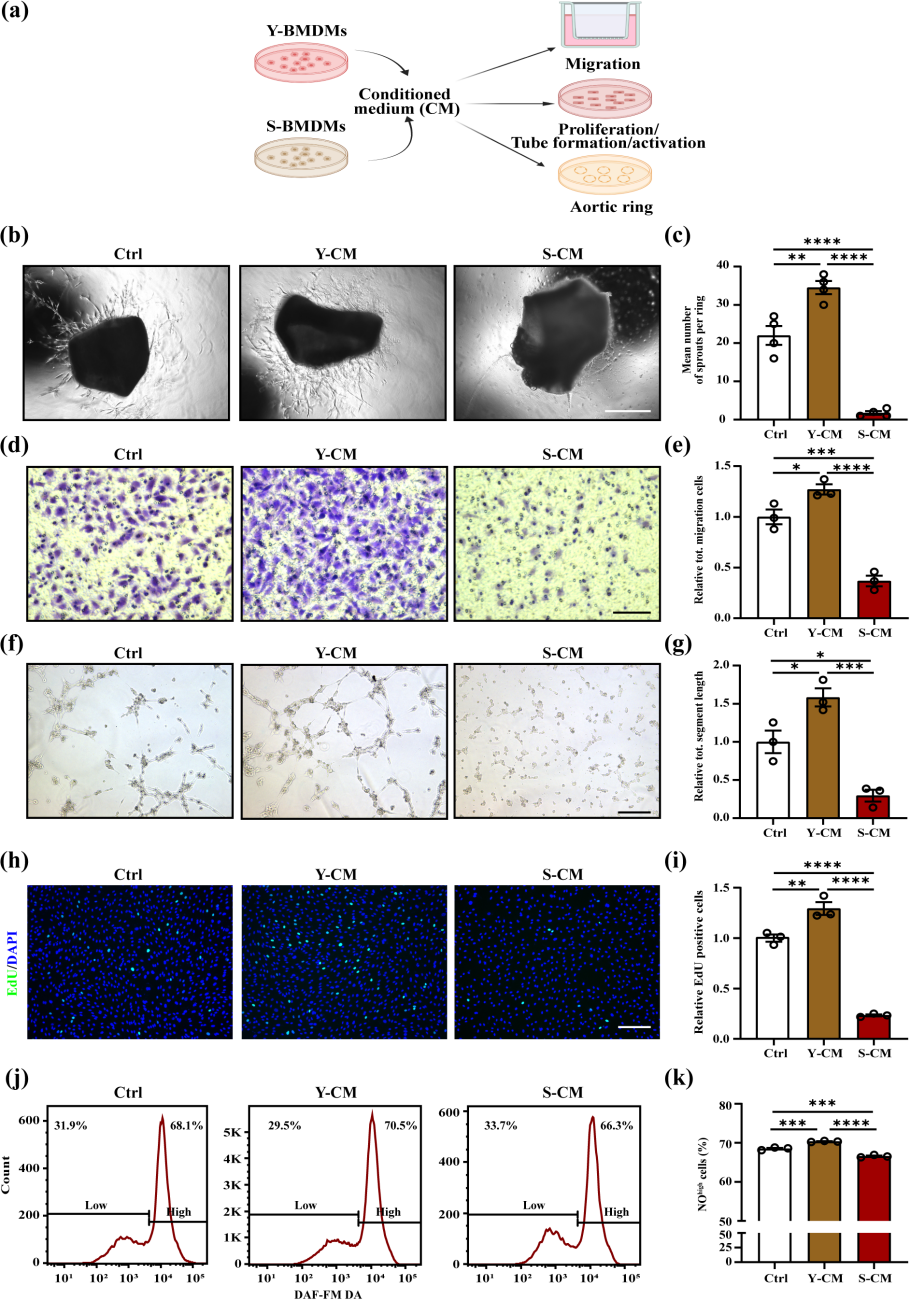
Conditioned media from senescent BMDMs actively affect the function of skeletal muscle ECs in vitro. (a) Scheme illustrating the experiment of co‐culture. (b, c) Representative images (b) and quantification (c) of sprouts of aortic rings treated with Y‐CM, S‐CM, or control media (*n* = 4; scale bar = 250 μm). (d, e) Representative images (d) and quantification (e) of transwell assays in mECs treated with Y‐CM, S‐CM, or control media (*n* = 3; scale bar = 75 μm). (f, g) Representative images (f) and quantification (g) of in vitro Matrigel assays in mECs treated with Y‐CM, S‐CM, or control media (*n* = 3; scale bar = 75 μm). (h, i) Representative images of EdU (green) and DAPI (blue) immunostaining (h) and quantification (i) of EdU^+^ cells in mECs treated with Y‐CM, S‐CM or control media (*n* = 3; scale bar = 75 μm). (j, k) Representative flow cytometric images (j) and quantification (k) of the percent of NO^high^ mECs treated with Y‐CM, S‐CM, or control media (*n* = 3). One‐way ANOVA. Error bars represent SEM. *, **, ***, **** Denote *p* < 0.05, *p* < 0.01, *p* < 0.001, and *p* < 0.0001, respectively.

To study whether SBMDMs affect ECs function ex vivo, we isolated aortic artery or primary skeletal muscle ECs (mECs) and stimulated them with CM from BMDMs cultures (Figure 3a). CM of SBMDMs decreased the sprouts of the aortic ring, while CM of YBMDMs increased (Figure 3b,c). The migration of mECs was significantly less upon S‐CM administration than Y‐CM (Figure 3d,e). Additionally, Matrigel assays and EdU staining showed S‐CM inhibited angiogenesis (Figure 3f,g) and proliferation (Figure 3h,i) of mECs compared with Y‐CM. Flow cytometry showed that S‐CM reduced NO expression of mECs, while Y‐CM promoted it (Figure 3j,k; Figure S4a,b). Grunewald et al. demonstrated soluble VEGFR1/FLT1 played a significant role in capillary rarefaction with age in the skeletal muscles (Grunewald et al. 2021). Our results showed that the mRNA levels of *Flt1* in mECs treated with S‐CM were significantly elevated compared with Y‐CM (Figure S4c). Together, these data indicated that senescent macrophages correlated closely with ECs dysfunction.

### 3.5 Senescent Macrophages‐Induced ECs Dysfunction Is Antiangiogenic VEGF‐A165B Dependent

To explore how senescent macrophages induce ECs dysfunction, we quantified the mRNA level of *Vegf‐a*, *Vegf‐a165a*, and *Vegf‐a165b* in BMDMs and SMPs. *Vegfa* mRNA expression was increased both in SBMDMs and SMPs compared to the young group. Moreover, proangiogenic *Vegf‐a165a* mRNA expression was not different between YBMDMs and SBMDMs or between non‐SMPs and SMPs. However, the antiangiogenic *Vegf‐a165b* mRNA expression was significantly higher in SBMDMs or SMPs (Figure S5a‐f). ELISA analysis of CM from BMDM cultures showed similar results (Figure S5g–i).

**FIGURE 4:**
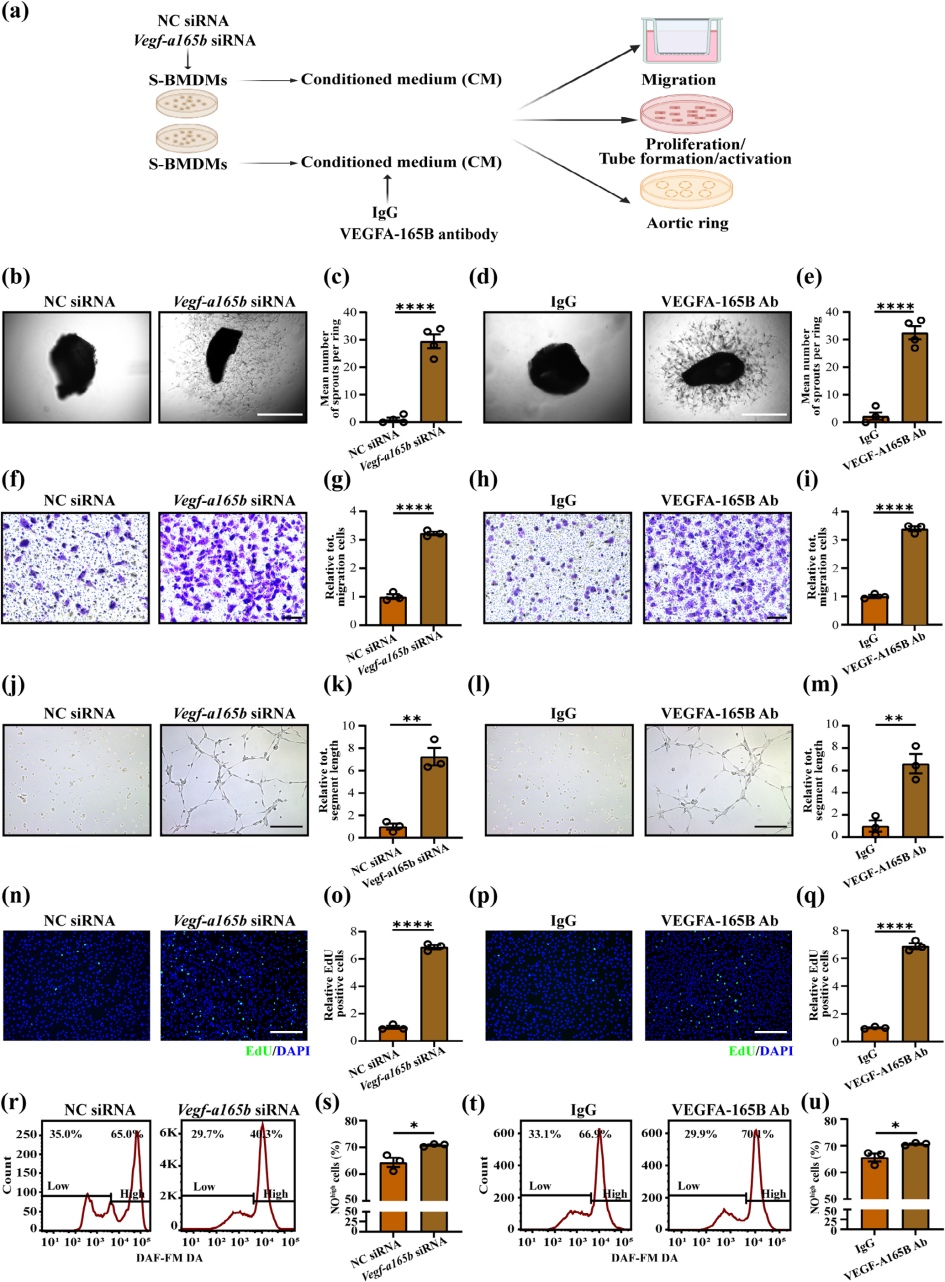
VEGF‐A165B mediates the effect of senescent BMDMs on the function of skeletal muscle ECs in vitro. (a) Scheme illustrating the experiment of co‐culture rescued by *Vegf‐a165b* siRNA or VEGF‐A165B antibody. (b, c) Representative images (b) and quantification (c) of sprouts of aortic rings in S‐CM treated with *Vegf‐a165b* siRNA or control siRNA (*n* = 4; scale bar = 250 μm). (d, e) Representative images (d) and quantification (e) of sprouts of aortic ring in S‐CM treated with isotype IgG or VEGF‐A165B antibody (*n* = 4; scale bar = 250 μm). (f, g) Representative images (f) and quantification (g) of transwell assays in mECs in S‐CM treated with *Vegf‐a165b* siRNA or control siRNA (*n* = 3; scale bar = 75 μm). (h, i) Representative images (h) and quantification (i) of transwell assays in mECs in S‐CM treated with isotype IgG or VEGF‐A165B antibody (*n* = 3; scale bar = 75 μm). (j, k) Representative images (j) and quantification (k) of in vitro Matrigel assays in mECs in S‐CM treated with *Vegf‐a165b siRNA* or control siRNA (*n* = 3; scale bar = 75 μm). (l, m) Representative images (l) and quantification (m) of in vitro Matrigel assays in mECs in S‐CM treated with isotype IgG or VEGF‐A165B antibody (*n* = 3; scale bar = 75 μm). (n, o) Representative images of EdU (green) and DAPI (blue) immunostaining (n) and quantification (o) of EdU^+^ cells in mECs in S‐CM treated with *Vegf‐a165b siRNA* or control siRNA (*n* = 3; scale bar = 75 μm). (p, q) Representative images of EdU (green) and DAPI (blue) immunostaining (p) and quantification (q) of EdU^+^ cells in mECs in S‐CM treated with isotype IgG or VEGF‐A165B antibody (*n* = 3; scale bar = 75 μm). (r, s) Representative flow cytometric images (r) and quantification (s) of the percent of NO^high^ mECs in S‐CM treated with *Vegf‐a165b siRNA* or control siRNA (*n* = 3). (t, u) Representative flow cytometric images (t) and quantification (u) of the percent of NO^high^ mECs in S‐CM treated with isotype IgG or VEGF‐A165B antibody (*n* = 3). Unpaired *t*‐tests. Error bars represent SEM. *, **, **** Denote *p* < 0.05, *p* < 0.01, and *p* < 0.0001, respectively.

In order to validate VEGF‐A165B mediates the effect of senescent macrophages on ECs function, aortic artery or mECs was treated with the CM from SBMDMs transfected with *Vegf‐a165b* siRNA or NC siRNA; or S‐CM added with VEGF‐A165B antibody or IgG (Figure 4a; Figure S6a,b). We found both *Vegf‐a165b* gene knockdown and VEGF‐A165B antibody neutralization could rescue the reduction of the sprouts of aortic ring (Figure 4b–e), and the impairment of migration (Figure 4f–i), tube formation (Figure 4j–m), proliferation (Figure 4n–q), and NO expression (Figure 4r–u; Figure S6c,d) of mECs induced by S‐CM. In summary, the data support that senescent macrophages affect ECs function partly via increasing VEGF‐A165B expression and secretion.

### 3.6 VEGF‐A165B Knockdown Rescues the Impaired Revascularization and ECs Dysfunction Induced by Senescent Macrophages in Young Mice Following Artery Occlusion

**FIGURE 5:**
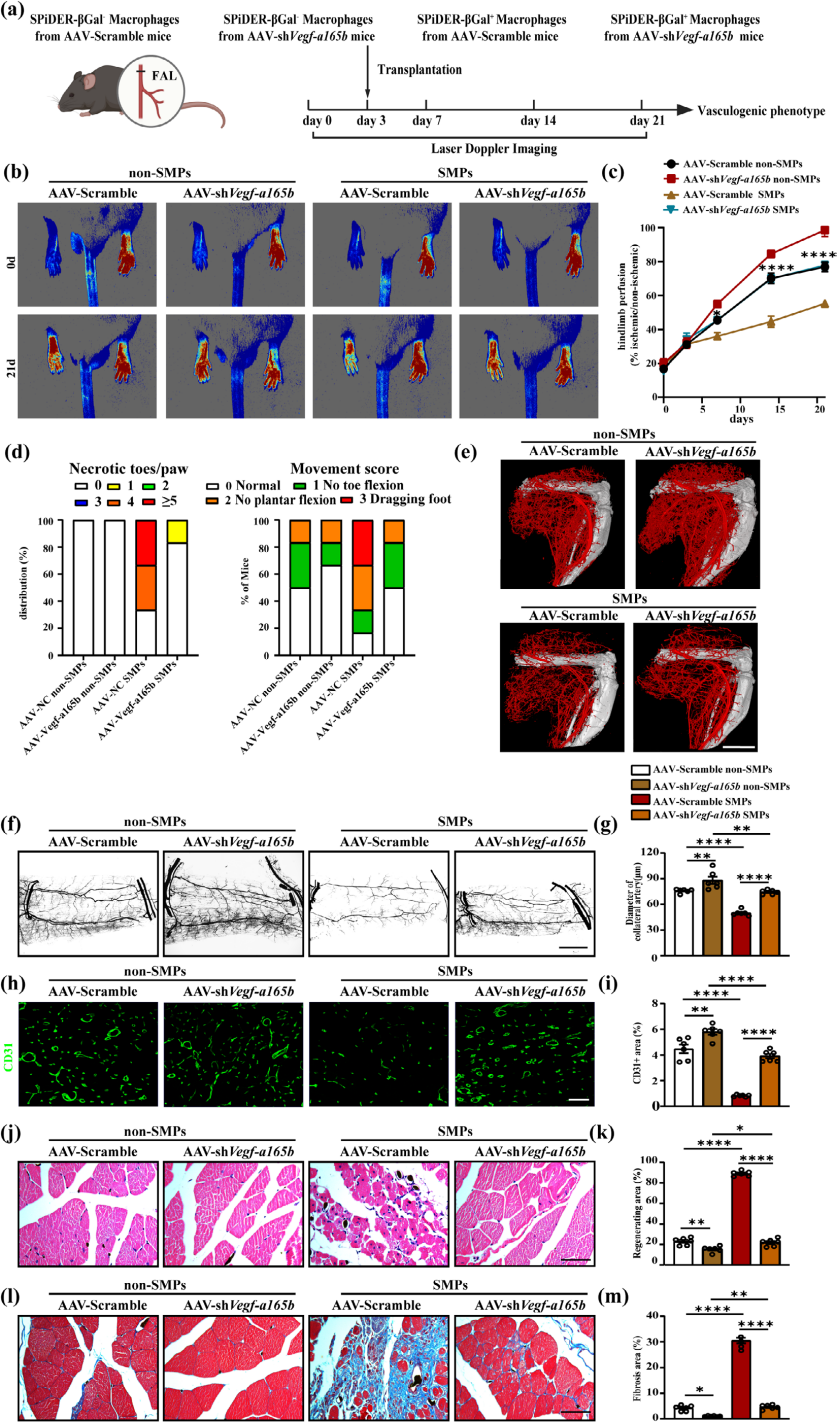
VEGF‐A165B knockdown rescues the effect of senescent macrophages on ischemia‐induced revascularization impairment. (a) Scheme showing transfer experiments of macrophages from mice treated with AAV‐sh*Vegf‐a165b*. (b, c) Representative image (b) and quantification (c) of hindlimb blood perfusion of mice transferred with SMPs or non‐SMPs from mice injected with AAV‐sh*Vegf‐a165b* or AAV‐Scramble before and after HLI surgery (*n* = 6). (d) Distribution of necrosis toes per paw (*n* = 6) and movement score (*n* = 6) of mice transferred with SMPs or non‐SMPs from mice injected with AAV‐sh*Vegf‐a165b* or AAV‐Scramble at 21 days after HLI. (e) Representative microCT images of hindlimb vasculature of mice transferred with SMPs or non‐SMPs from mice injected with AAV‐sh*Vegf‐a165b* or AAV‐Scramble at 7 days after HLI (*n* = 6; scale bar = 4 mm). (f, g) Representative whole mount images (f) and quantification (g) of Microfil‐filled gracilis muscle vasculature of mice transferred with SMPs or non‐SMPs from mice injected with AAV‐sh*Vegf‐a165b* or AAV‐Scramble from day 21 post‐HLI hindlimbs (*n* = 6; scale bar = 100 μm). (h, i) Representative CD31 immunofluorescent images (h) and quantification (i) on gastrocnemius cross sections of mice transferred with SMPs or non‐SMPs from mice injected with AAV‐sh*Vegf‐a165b* or AAV‐Scramble at 21 days after HLI (*n* = 6; scale bar = 50 μm). (j, k) Representative HE staining images (j) and quantification (k) of the regenerating area on gastrocnemius cross sections of mice transferred with SMPs or non‐SMPs from mice injected with AAV‐sh*Vegf‐a165b* or AAV‐NC at 21 days after HLI (*n* = 6; scale bar = 75 μm). (l, m) Representative Masson staining images(l) and quantification (m) of fibrosis area on gastrocnemius cross sections of mice transferred with SMPs or non‐SMPs from mice injected with AAV‐sh*Vegf‐a165b* or AAV‐Scramble at 21 days after HLI (*n* = 6; scale bar = 75 μm). Laser Doppler imaging experiments were analyzed using two‐way ANOVA; other experiments were analyzed using one‐way ANOVA. Error bars represent SEM. *, **, **** Denote *p* < 0.05, *p* < 0.01, and *p* < 0.0001, respectively.

Furthermore, we investigated whether VEGFA‐165B mediated the role of senescent macrophages in revascularization and ECs function in mice following artery occlusion. We sorted non‐SMPs or SMPs in ischemic skeletal muscle of old mice injected with AAV‐Scramble and AAV‐sh*Vegf‐a165b* based on the SPiDER‐β‐gal, and injected either AAV‐Scramble non‐SMPs, AAV‐sh*Vegf‐a165b* non‐SMPs, AAV‐Scramble SMPs, or AAV‐sh*Vegf‐a165b* SMPs into the hindlimb of young mice 3 days after HLI (Figure 5a; Figure S7a,b). Laser Doppler imaging showed that hindlimb perfusion was improved in the AAV‐sh*Vegf‐a165b* non‐SMPs group. Also, the transfer of AAV‐sh*Vegf‐a165b* SMPs improved hindlimb perfusion, which was similar to the group transferred with AAV‐Scramble non‐SMPs (Figure 5b,c). Meanwhile, the transfer of AAV‐sh*Vegf‐a165b* SMPs displayed a lower frequency of necrotic toes and a higher score of movement (Figure 5d). Moreover, microCT of the hindlimb (Figure 5e), whole‐mounted gracilis (Figure 5f,g) and immunofluorescence (Figure 5h,i) showed that the transfer of SMPs from mice injected with AAV‐sh*Vegf‐a165b* rescued the reduction of vascular density, the diameter of the preexisting collateral artery, and capillary density. Alongside, AAV‐sh*Vegf‐a165b* SMPs transfer inhibited muscle regeneration (Figure 5j,k) and fibrosis (Figure 5l,m).

In addition, AAV‐sh*Vegf‐a165b* SMPs transfer activated arterial (Figure S7c,d), capillary ECs (Figure S7e,f) proliferation and p‐eNOS/NO signaling (Figure S7g–i) in ischemic hindlimb muscle. Thus, VEGF‐A165B mediated the effect of senescent macrophages in age‐induced muscle revascularization impairment and ECs dysfunction.

### 3.7 Plasma VEGF‐A165B Is Elevated in Old Patients With PAD and Positively Correlated With the Severity of PAD

**FIGURE 6:**
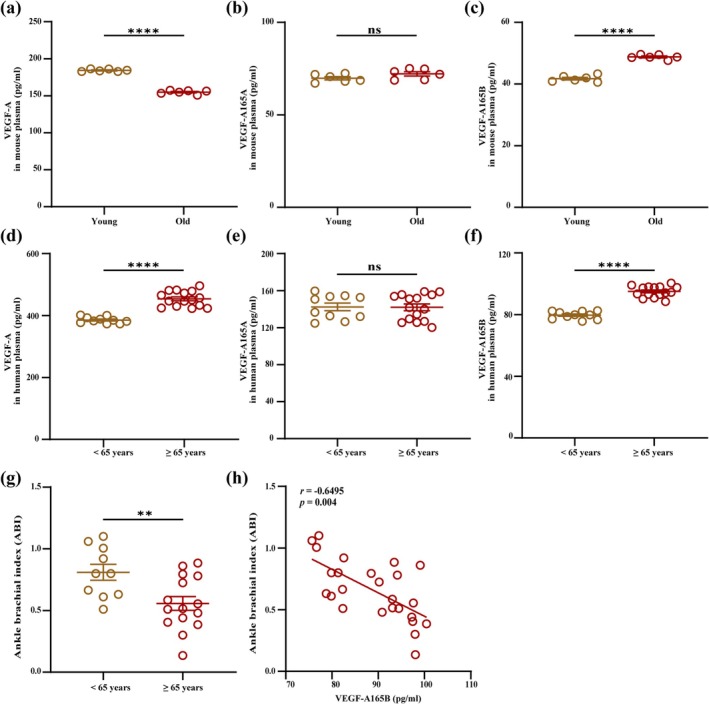
Plasma VEGF‐A165B is elevated in old patients with PAD and positively correlated with the severity of PAD. (a–c) ELISA analysis of VEGF‐A (a), VEGF‐A165A (b) and VEGF‐A165B (c) protein levels in plasma from young and old mice at 7 days following HLI (*n* = 6). (d–f) ELISA analysis of VEGF‐A (d), VEGF‐A165A (e) and VEGF‐A165B (f) protein levels in plasma from young (< 65 years) and old (≥ 65 years) patients with PAD (Young group, *n* = 10; Old group, *n* = 15). (g) ABI analysis between young (< 65 years) and old (≥ 65 years) patients with PAD (Young group, *n* = 10; Old group, *n* = 15). (h) Correlation analysis between plasma VEGF‐A165B level and ABI (*n* = 25). Unpaired *t*‐tests. Error bars represent SEM. **, ****, and ns denote *p* < 0.01, *p* < 0.0001, and not significant, respectively. [Correction added on 26 April 2025 after first online publication: The Y‐axis label of Figure 6c has been corrected from “VEGF‐A165A” to “VEGF‐A165B.”]

In the HLI model, we found that plasma VEGF‐A and VEGF‐A165B protein levels were increased in old mice . While plasma VEGF‐A165A concentration was not significantly different between young and old mice (Figure 6a‐c). Meanwhile, we performed an ELISA assay to assess the clinical relevance of VEGF‐A, VEGF‐A165A, and VEGF‐A165B to PAD in old patients. Plasma VEGF‐A and VEGF‐A165B concentrations were significantly higher in old patients with PAD (Age ≥ 65 years) than those in the young group (Age < 65 years), and VEGF‐A165A protein levels were similar between them (Figure 6d‐f). Further, our data showed that old patients with PAD had lower ABI compared with the young group (Figure 6g). Correlation analysis indicated that VEGF‐A165B was positively correlated with a lower ABI (Figure 6h).

## 4 Discussion

To our knowledge, our research firstly identified that macrophages were more senescent in the ischemic skeletal muscle of old mice. We demonstrated that senescent macrophages inhibited revascularization via increasing VEGF‐A165B expression and secretion. Additionally, plasma VEGF‐A165B protein levels were upregulated in the old patients with PAD, which is positively correlated with the severity of PAD.

Macrophages are essential immune cells involved in various physiological and pathological processes that maintain homeostasis (Lazarov et al. 2023). While numerous studies have examined the changes in macrophages during healthy aging, including the loss of splenic marginal macrophages and the altered interaction with other immune cells (Birjandi et al. 2011), the functional changes in macrophages within damaged aging tissues remain underexplored (Fontana et al. 2013; Lumeng et al. 2011; Gomez et al. 2007). Previous research has shown delayed muscle and bone repair in elderly humans and mice, primarily due to altered chemokine signaling, leading to reduced macrophage infiltration or dysfunctional recruited macrophages (Shavlakadze et al. 2010; Greiwe et al. 2001; Wang et al. 2018; Clark et al. 2020; Larsen et al. 2017). Recently, Cai et al. found using single‐cell RNA‐seq that *Arg1* ^+^ macrophages were activated in young muscle but suppressed in aged regenerating muscle based on a cryoinjury model. These *Arg1* ^+^ macrophages were positively associated with ECs proliferation, vascular endothelial growth factor production, wound healing, and regulation of angiogenesis (Cai et al. 2023). However, in this study, we discovered through RNA‐seq that the *Arg1* expression in ischemic skeletal muscle macrophages was not significantly downregulated in old mice, but the expression of senescent and proinflammatory genes was highly elevated. This may be attributed to the differences in animal models, as well as the bulk RNA‐seq having insufficient sequencing depth compared to single‐cell RNA‐seq. To further explore the senescent characteristics of macrophages, we showed through flow cytometry analysis that there was a significant upregulation of the senescent marker SPiDER‐β‐gal in both total macrophages and CD86^+^ or CD206^+^ macrophages in the ischemic muscles of old mice after HLI. To elucidate the role of senescent macrophages in revascularization, we used an immune cell transplantation model. Senescent macrophages isolated from ischemic muscles after HLI were transplanted into young mice, resulting in a significant inhibition of revascularization, ECs proliferation, and activation. These findings suggest that senescent macrophages contribute to age‐related revascularization impairment.

Macrophages are crucial participants in revascularization, playing a complex role in regulating ECs (Wynn and Vannella 2016). Macrophages induce extracellular matrix remodeling and regulate ECs functions through the paracrine secretion of matrix metalloproteinases (MMPs) (Shireman 2007; Dodd et al. 2011) and various proangiogenic growth factors, including VEGF‐A and fibroblast growth factor 2 (FGF2) (Spiller et al. 2014; Morrison et al. 2014; Fantin et al. 2010). To investigate the effects of senescent macrophages on endothelial function in vitro, we established a model of senescent macrophages using BMDMs. After treating aortic rings or ECs with CM from BMDMs, we observed that senescent BMDMs inhibited aortic sprouting, endothelial tube formation, migration, proliferation, and activation. These findings indicate that senescent macrophages impair endothelial function through paracrine mechanisms.

It is well established that VEGF‐A plays a critical role in promoting revascularization, with macrophages being the primary cells producing VEGF‐A (Pérez‐Gutiérrez and Ferrara 2023; Willenborg et al. 2012). VEGF‐A has been considered a potential therapy for promoting angiogenesis in clinical PAD, but numerous clinical studies were terminated for various reasons. Limitations include preclinical models predominantly focusing on young individuals, limiting the applicability of study findings to old populations (Cooke and Losordo 2015). Moreover, elderly patients often face challenges such as declining liver and kidney function, which can impact pharmacokinetics and efficacy assessments in preclinical research (Shenoy and Harugeri 2015). Research suggests that VEGF‐A can produce the antiangiogenic splice variant VEGF‐A165B, which inhibits revascularization (Kikuchi et al. 2014; Ganta et al. 2017; Ganta et al. 2019). Studies have reported a close association between abnormal splicing events and aging (Lee et al. 2016; Li et al. 2017). Changes in the selective splicing of genes such as *Abcr*, *Eaat2*, *Eeg*, and *Tp53* have been confirmed to be related to aging and age‐related diseases (Lin et al. 1998; Tang et al. 2013; Allikmets et al. 1997; Blanco et al. 2008). Therapies aimed at correcting splicing errors are actively being researched (Kole et al. 2012). However, there have been no reports on how aging affects VEGF‐A splicing. We supplement the understanding of VEGF‐A splicing changes during aging, thereby enriching the research on age‐related alternative splicing of genes. In this study, we found that both primary senescent macrophages and senescent BMDMs exhibited significantly increased mRNA levels of *Vegf‐a* and *Vegf‐a165b*, but not *Vegf‐a165a* compared to young mice. Additionally, ELISA results showed that the CM from senescent BMDMs had significantly higher levels of VEGF‐A and VEGF‐A165B proteins compared with the young group, while VEGF‐A165A protein levels remained unchanged. These findings suggest that senescent macrophages may inhibit endothelial function and impair revascularization through the high expression and secretion of the VEGF‐A splice variant VEGF‐A165B.

Skeletal muscle is a highly vascularized tissue with remarkable regenerative capacity (Almada and Wagers 2016; Zhang et al. 2020). Muscle regeneration relies on the precisely timed interactions and coordination among various cells within the muscle microenvironment, including muscle stem cells, ECs, and macrophages (Zhang et al. 2020; Yin et al. 2013). Revascularization is crucial during muscle regeneration, which not only restores the supply of oxygen and nutrients to the regenerating muscle tissue but also enhances the proliferation of muscle progenitor cells through secreting growth factors (Krishnasamy et al. 2017; Potente et al. 2011; Borselli et al. 2010). Macrophages could influence muscle regeneration by shifting their polarization state to regulate revascularization (He et al. 2012; Zhang et al. 2020). At the onset of ischemia, macrophages take on a proinflammatory phenotype, but soon transition to an anti‐inflammatory phenotype, which actively facilitates revascularization and muscle regeneration (Arnold et al. 2007). It is worth noting that a study showed that increased VEGF‐A165B expression in macrophages induces a proinflammatory phenotype that directly impairs angiogenesis (Ganta et al. 2019). What's more, macrophages could impact muscle regeneration by secreting inflammatory cytokines and growth factors, such as interleukin‐10 (IL‐10), insulin growth factor‐1 (IGF‐1), and VEGF‐A (Zhang et al. 2020; Dort et al. 2019; Borselli et al. 2010). In our study, we found senescent macrophages could inhibit muscle regeneration by VEGF‐A165B signaling. However, it remains to be investigated whether the VEGF‐A165B secreted by senescent macrophages inhibits muscle regeneration by influencing ECs, modulating the polarization state of macrophages, or directly targeting muscle cells.

While studies have identified significantly elevated levels of VEGF‐A165B in the plasma of PAD patients (Kikuchi et al. 2014), the differential expression of VEGF‐A165B in the plasma of young and old PAD patients has not been reported. Initially, we examined the expression of VEGF‐A and its splice variants in the plasma of young and old mice after ischemia. We found that compared with the young group, the plasma levels of VEGF‐A and VEGF‐A165B were significantly upregulated in old mice, while VEGF‐A165A levels remained unchanged. Similarly, higher levels of VEGF‐A and VEGF‐A165B were observed in the plasma of old PAD patients, with no significant difference in VEGF‐A165A levels. Furthermore, plasma VEGF‐A165B levels were positively correlated with lower ABI.

Although we found that senescent macrophages contribute to age‐related revascularization impairment, their causal relationship needs to be confirmed using genetic mouse models. Additionally, we observed that senescent macrophages highly express VEGF‐A165B, but the upstream molecular mechanisms regulating VEGF‐A165B during aging require further investigation. Furthermore, in this study, we propose that the secretion of VEGF‐A165B by senescent macrophages mediates endothelial dysfunction and revascularization impairment. It remains to be explored whether other secretions from macrophages, such as the SASP‐related markers *Icam1* and *Cxcl12* mentioned earlier, are also involved. Although previous studies have demonstrated gender was a key determinant in epidemiology, differential outcomes, and proposed biological mechanisms of PAD (Morrison and Aday 2022; Pabon et al. 2022), this study did not stratify by gender due to limited sample size of female patients with PAD. In the future, we need to include more female patients with PAD to substantiate our results.

In conclusion, to our knowledge, this study is the first to identify the significant role of senescent macrophages in age‐related revascularization impairment. The potential mechanism involves the high expression of the antiangiogenic isoform VEGF‐A165B by senescent macrophages, leading to endothelial dysfunction. This research aims to address the limitations of current preclinical models by better reflecting the clinical reality of patients and suggests that targeting senescent macrophages may be a promising direction for PAD clinical treatment.

## Author Contributions

Conceptualization: Qun Huang. Investigation: Minghong Chen, Junyu Chen, Yu Liu, Xuerui Wang, Meilian Yao, Jing Chen, and Jian Zhang. Writing: Minghong Chen, Junyu Chen, and Qun Huang.

## Conflicts of Interest

The authors declare no conflicts of interest.

## Supporting information


**Figure S1.** Aging promotes the macrophages senescence in hindlimb skeletal muscle. Gating strategy of macrophages in the hindlimb skeletal muscle. Representative flow cytometry plots for macrophages of hindlimb skeletal muscle stained with CD86, blank control and fluorescence minus one (FMO) control. Representative flow cytometry plots for macrophages of hindlimb skeletal muscle stained with CD206, blank control and FMO control. (d, g) Representative flow cytometry plots (d) and quantification (g) of the percent of SPiDER‐βGal^+^F4/80^+^ cells in the hindlimb skeletal muscle (*n* = 6). (e, h) Representative flow cytometry plots (e) and quantification (h) of the percent of SPiDER‐βGal^+^CD86^+^ cells in the hindlimb skeletal muscle (*n* = 6). (f, i) Representative flow cytometry plots (f) and quantification (i) of the percent of SPiDER‐βGal^+^CD206^+^ cells in the hindlimb skeletal muscle (*n* = 6). Representative flow cytometry plots for macrophages of hindlimb skeletal muscle stained with SPiDER‐βGal, blank control and FMO control. Immunoblot images and quantification for MHCII or LYVE1 protein levels in non‐SMPs and SMPs isolated from hindlimb skeletal muscle of 24‐month‐old mice (*n* = 3). (l, m) Real‐time PCR analysis of *Mhcii* (l) and *Lyve1* (m) in non‐SMPs and SMPs isolated from hindlimb skeletal muscle of 24‐month‐old mice (*n* = 3). Unpaired *t*‐tests. Error bars represent SEM. **, ***, **** and ns denote *p* < 0.01, *p* < 0.001, and *p* < 0.0001, not significant, respectively.
**Figure S2.** Senescent macrophages actively affect proliferation and eNOS phosphorylation of skeletal muscle ECs in vivo. (a, b) Representative CD31 (green), aSMA (red), and KI67 (gray) immunofluorescent images (a) and quantification (b) on gastrocnemius cross sections of mice transferred with SMPs or non‐SMPs at 7 days after HLI (*n* = 6; scale bar = 50 μm). (c, d) Representative CD31 (green) and KI67 (red) immunofluorescent images (c) and quantification (d) on gastrocnemius cross sections of mice transferred with SMPs or non‐SMPs at 7 days after HLI (*n* = 6; scale bar = 20 μm). (e, f) Representative phospho‐eNOS immunohistochemical images (e) and quantification (f) on gastrocnemius cross sections of mice transferred with SMPs or non‐SMPs at 7 days after HLI (*n* = 6; scale bar = 100 μm). (g) NO levels in ischemic hindlimb muscle ECs of mice transferred with SMPs or non‐SMPs at 7 days (*n* = 3). One‐way analysis of variance (ANOVA). Error bars represent SEM. ***, **** and ns denote *p* < 0.001, *p* < 0.0001 and not significant, respectively.
**Figure S3.** Features of senescent macrophages in vitro are replicated in vivo. Scheme showing the experiment of senescent BMDM model. Representative F4/80 immunofluorescent images of YBMDMs and SBMDMs (*n* = 3; scale bar = 20 μm). (c, d) Representative flow cytometry plots (c) and quantification (d) of the percent of F4/80^+^ cells (*n* = 3). (e, f) Representative images (c) and quantification (d) of SA‐β‐gal staining positive macrophages (*n* = 3; scale bar = 75 μm). Immunoblot images and quantification for P21 or LMNB1 protein levels in YBMDMs and SBMDMs (*n* = 3). Immunoblot images and quantification for MHCII or LYVE1 protein levels in YBMDMs and SBMDMs (*n* = 3). (i, j) Representative images of KI67 (red) and DAPI (blue) immunostaining (i) and quantification (j) of KI67^+^ cells in YBMDMs and SBMDMs (*n* = 3; scale bar = 20 μm). (k, l) Representative images of γ‐H2AX (red) and DAPI (blue) immunostaining (k) and quantification (l) of γ‐H2AX^+^ cells in YBMDMs and SBMDMs (*n* = 3; scale bar = 20 μm). (m) The heatmap of real‐time PCR analysis for SASP markers, senescence markers, and Sirt family in YBMDMs and SBMDMs (*n* = 3). (n–s) Real‐time PCR analysis of SASP markers (n), senescence markers (o), Sirt family (p), pro‐ or anti‐inflammatory markers (q), *Mhcii* (r) and *Lyve1* (s) in YBMDMs and SBMDMs (*n* = 3). Real‐time PCR experiment of pro‐ or anti‐inflammatory markers was analyzed by one‐way analysis of variance (ANOVA), others were analyzed by Unpaired *t*‐tests. Error bars represent SEM. *, **, ***, **** Denote *p* < 0.05, *p* < 0.01, *p* < 0.001, and *p* < 0.0001, respectively.
**Figure S4.** DAF‐FM DA staining control and Flt1 mRNA levels in mECs. Representative flow cytometry plots for mECs with blank control and DAF‐FM DA. Quantification of the percent of NO^low^ mECs treated with Y‐CM, S‐CM or control media (*n* = 3). Real‐time PCR analysis of *Flt1* in mECs treated with Y‐CM, S‐CM and control media (*n* = 3). One‐way analysis of variance (ANOVA). Error bars represent SEM. * and ns denote *p* < 0.05 and not significant, respectively.
**Figure S5.** Senescent macrophages highly express VEGF‐A165B in vivo and in vitro. (a–c) Real‐time PCR analysis of *Vegf‐a* (a), *Vegf‐a165a* (b) and *Vegf‐a165b* (c) expression in young and senescent BMDMs (*n* = 6). (d–f) Real‐time PCR analysis of *Vegfa* (d), *Vegf‐a165a* (e) and *Vegf‐a165b* (f) expression in non‐SMPs and SMPS of ischemic hindlimb muscle (*n* = 6). (g–i) ELISA analysis of VEGF‐A (g), VEGF‐A165A (h) and VEGF‐A165B (i) protein levels in conditional media from young and senescent BMDMs (*n* = 6). Unpaired *t*‐tests. Error bars represent SEM. *, ** denote *p* < 0.05, and *p* < 0.01, respectively.
**Figure S6.** Knockdown efficiency of VEGF‐A165B in senescent BMDMs. Immunoblot images and quantification for VEGF‐A165B protein levels in SBMDMs treated with *Vegf‐a165b siRNA* or control siRNA (*n* = 3). Real‐time PCR analysis of *Vegf‐a165b* expression in SBMDMs treated with *Vegf‐a165b siRNA* or control siRNA (*n* = 3). Quantification of the percent of NO^low^ mECs in S‐CM treated with control siRNA or *Vegf‐a165b siRNA* (*n* = 3). Quantification of the percent of NO^low^ mECs in S‐CM treated with isotype IgG or VEGF‐A165B antibody (*n* = 3). Unpaired *t*‐tests. Error bars represent SEM. ** Denote *p* < 0.01.
**Figure S7.** VEGF‐A165B knockdown rescue the effect of senescent macrophages on proliferation and eNOS phosphorylation of skeletal muscle ECs in vivo. Immunoblot images and quantification for VEGF‐A165B protein levels in hindlimb skeletal muscle macrophages of mice injected with AAV‐sh*Vegf‐a165b* or AAV‐Scramble (*n* = 3). Real‐time PCR analysis of *Vegf‐a165b* expression in hindlimb skeletal muscle macrophages of mice injected with AAV‐sh*Vegf‐a165b* or AAV‐Scramble (*n* = 3). (c, d) Representative CD31 (green), aSMA (red), and KI67 (gray) immunofluorescent images (c) and quantification (d) on gastrocnemius cross sections of mice transferred with SMPs or non‐SMPs from mice injected with AAV‐sh*Vegf‐a165b* or AAV‐Scramble at 7 days after HLI (*n* = 6; scale bar = 50 μm). (e, f) Representative CD31 (green) and KI67 (red) immunofluorescent images (e) and quantification (f) on gastrocnemius cross sections of mice transferred with SMPs or non‐SMPs from mice injected with AAV‐sh*Vegf‐a165b* or AAV‐Scramble at 7 days after HLI (*n* = 6; scale bar = 20 μm). (g, h) Representative phospho‐eNOS immunohistochemical images (g) and quantification (h) on gastrocnemius cross sections of mice transferred with SMPs or non‐SMPs from mice injected with AAV‐sh*Vegf‐a165b* or AAV‐Scramble at 7 days after HLI (*n* = 6; scale bar = 100 μm). (i) NO levels in ischemic hindlimb muscle ECs of mice transferred with SMPs or non‐SMPs from mice injected with AAV‐sh*Vegf‐a165b* or AAV‐Scramble at 7 days (*n* = 3). Immunoblot and Real‐time PCR experiments of Vegf‐a165b levels in hindlimb skeletal muscle macrophages was analyzed by unpaired *t*‐tests, others were analyzed by one‐way analysis of variance (ANOVA). Error bars represent SEM. ***, **** and ns denote *p* < 0.001, 0.0001 and not significant, respectively.

Table S1.

Table S2.

Table S3.

## Data Availability

The data that support the findings of this study are available from the corresponding author upon reasonable request.

## References

[acel70059-bib-0001] Ahmadi, M. , A. Karlsen , J. Mehling , C. Soendenbroe , A. L. Mackey , and R. D. Hyldahl . 2022. “Aging Is Associated With an Altered Macrophage Response During Human Skeletal Muscle Regeneration.” Experimental Gerontology 169: 111974. 10.1016/j.exger.2022.111974.. 36228835

[acel70059-bib-0002] Allikmets, R. , N. F. Shroyer , N. Singh , et al. 1997. “Mutation of the Stargardt Disease Gene (ABCR) in Age‐Related Macular Degeneration.” Science 277: 1805–1807. 10.1126/science.277.5333.1805.. 9295268

[acel70059-bib-0003] Almada, A. E. , and A. J. Wagers . 2016. “Molecular Circuitry of Stem Cell Fate in Skeletal Muscle Regeneration, Ageing and Disease.” Nature Reviews. Molecular Cell Biology 17: 267–279. 10.1038/nrm.2016.7.. 26956195 PMC4918817

[acel70059-bib-0004] Annex, B. H. , and J. P. Cooke . 2021. “New Directions in Therapeutic Angiogenesis and Arteriogenesis in Peripheral Arterial Disease.” Circulation Research 128: 1944–1957. 10.1161/CIRCRESAHA.121.318266.. 34110899 PMC8538391

[acel70059-bib-0006] Arnold, L. , A. Henry , F. Poron , et al. 2007. “Inflammatory Monocytes Recruited After Skeletal Muscle Injury Switch Into Antiinflammatory Macrophages to Support Myogenesis.” Journal of Experimental Medicine 204, no. 5: 1057–1069. 10.1084/jem.20070075. 17485518 PMC2118577

[acel70059-bib-0007] Arras, M. , W. D. Ito , D. Scholz , B. Winkler , J. Schaper , and W. Schaper . 1998. “Monocyte Activation in Angiogenesis and Collateral Growth in the Rabbit Hindlimb.” Journal of Clinical Investigation 101: 40–50. 10.1172/JCI119877.. 9421464 PMC508538

[acel70059-bib-0008] Assouvie, A. , L. P. Daley‐Bauer , and G. Rousselet . 2018. “Growing Murine Bone Marrow‐Derived Macrophages.” Methods in Molecular Biology 1784: 29–33. 10.1007/978-1-4939-7837-3_3.. 29761385

[acel70059-bib-0009] Bannon, P. , S. Wood , T. Restivo , L. Campbell , M. J. Hardman , and K. A. Mace . 2013. “Diabetes Induces Stable Intrinsic Changes to Myeloid Cells That Contribute to Chronic Inflammation During Wound Healing in Mice.” Disease Models & Mechanisms 6: 1434–1447. 10.1242/dmm.012237.. 24057002 PMC3820266

[acel70059-bib-0010] Becker, L. , L. Nguyen , J. Gill , S. Kulkarni , P. J. Pasricha , and A. Habtezion . 2018. “Age‐Dependent Shift in Macrophage Polarisation Causes Inflammation‐Mediated Degeneration of Enteric Nervous System.” Gut 67: 827–836. 10.1136/gutjnl-2016-312940.. 28228489 PMC5565713

[acel70059-bib-0011] Birjandi, S. Z. , J. A. Ippolito , A. K. Ramadorai , and P. L. Witte . 2011. “Alterations in Marginal Zone Macrophages and Marginal Zone B Cells in Old Mice.” Journal of Immunology 186: 3441–3451. 10.4049/jimmunol.1001271.. PMC3420341 21307289

[acel70059-bib-0012] Blacher, E. , C. Tsai , L. Litichevskiy , et al. 2022. “Aging Disrupts Circadian Gene Regulation and Function in Macrophages.” Nature Immunology 23: 229–236. 10.1038/s41590-021-01083-0.. 34949832 PMC9704320

[acel70059-bib-0013] Blanco, F. J. , M. T. Grande , C. Langa , et al. 2008. “S‐Endoglin Expression Is Induced in Senescent Endothelial Cells and Contributes to Vascular Pathology.” Circulation Research 103: 1383–1392. 10.1161/CIRCRESAHA.108.176552.. 18974388

[acel70059-bib-0014] Borselli, C. , H. Storrie , F. Benesch‐Lee , et al. 2010. “Functional Muscle Regeneration With Combined Delivery of Angiogenesis and Myogenesis Factors.” Proceedings of the National Academy of Sciences of the United States of America 107: 3287–3292. 10.1073/pnas.0903875106.. 19966309 PMC2840452

[acel70059-bib-0015] Cai, Y. , M. Xiong , Z. Xin , et al. 2023. “Decoding Aging‐Dependent Regenerative Decline Across Tissues at Single‐Cell Resolution.” Cell Stem Cell 30: 1674–1691.e8. 10.1016/j.stem.2023.09.014.. 37898124

[acel70059-bib-0016] Chalothorn, D. , J. A. Clayton , H. Zhang , D. Pomp , and J. E. Faber . 2007. “Collateral Density, Remodeling, and VEGF‐A Expression Differ Widely Between Mouse Strains.” Physiological Genomics 30: 179–191. 10.1152/physiolgenomics.00047.2007.. 17426116

[acel70059-bib-0017] Childs, B. G. , D. J. Baker , T. Wijshake , C. A. Conover , J. Campisi , and J. M. van Deursen . 2016. “Senescent Intimal Foam Cells Are Deleterious at all Stages of Atherosclerosis.” Science 354: 472–477. 10.1126/science.aaf6659.. 27789842 PMC5112585

[acel70059-bib-0018] Clark, D. , S. Brazina , F. Yang , et al. 2020. “Age‐Related Changes to Macrophages Are Detrimental to Fracture Healing in Mice.” Aging Cell 19: e13112. 10.1111/acel.13112.. 32096907 PMC7059136

[acel70059-bib-0019] Cooke, J. P. , and D. W. Losordo . 2015. “Modulating the Vascular Response to Limb Ischemia.” Circulation Research 116: 1561–1578. 10.1161/CIRCRESAHA.115.303565.. 25908729 PMC4869986

[acel70059-bib-0020] Cui, C.‐Y. , R. K. Driscoll , Y. Piao , C. W. Chia , M. Gorospe , and L. Ferrucci . 2019. “Skewed Macrophage Polarization in Aging Skeletal Muscle.” Aging Cell 18: e13032. 10.1111/acel.13032.. 31478346 PMC6826159

[acel70059-bib-0021] Dodd, T. , R. Jadhav , L. Wiggins , et al. 2011. “MMPs 2 and 9 Are Essential for Coronary Collateral Growth and Are Prominently Regulated by p38 MAPK.” Journal of Molecular and Cellular Cardiology 51: 1015–1025. 10.1016/j.yjmcc.2011.08.012.. 21884701 PMC3208797

[acel70059-bib-0022] Dort, J. , P. Fabre , T. Molina , and N. A. Dumont . 2019. “Macrophages Are Key Regulators of Stem Cells During Skeletal Muscle Regeneration and Diseases.” Stem Cells International 2019: 4761427. 10.1155/2019/4761427.. 31396285 PMC6664695

[acel70059-bib-0023] Dungan, C. M. , K. A. Murach , C. J. Zdunek , et al. 2022. “Deletion of SA β‐Gal+ Cells Using Senolytics Improves Muscle Regeneration in Old Mice.” Aging Cell 21: e13528. 10.1111/acel.13528.. 34904366 PMC8761017

[acel70059-bib-0024] Duong, L. , H. Radley , B. Lee , et al. 2021. “Macrophage Function in the Elderly and Impact on Injury Repair and Cancer.” Immunity & Ageing 18: 4. 10.1186/s12979-021-00215-2.. 33441138 PMC7805172

[acel70059-bib-0025] Epstein, S. E. , R. M. Lassance‐Soares , J. E. Faber , and M. S. Burnett . 2012. “Effects of Aging on the Collateral Circulation, and Therapeutic Implications.” Circulation 125: 3211–3219. 10.1161/CIRCULATIONAHA.111.079038.. 22733335

[acel70059-bib-0026] Faber, J. E. , H. Zhang , R. M. Lassance‐Soares , et al. 2011. “Aging Causes Collateral Rarefaction and Increased Severity of Ischemic Injury in Multiple Tissues.” Arteriosclerosis, Thrombosis, and Vascular Biology 31: 1748–1756. 10.1161/ATVBAHA.111.227314.. 21617137 PMC3141082

[acel70059-bib-0027] Fantin, A. , J. M. Vieira , G. Gestri , et al. 2010. “Tissue Macrophages Act as Cellular Chaperones for Vascular Anastomosis Downstream of VEGF‐Mediated Endothelial Tip Cell Induction.” Blood 116: 829–840. 10.1182/blood-2009-12-257832.. 20404134 PMC2938310

[acel70059-bib-0028] Fontana, L. , E. Zhao , M. Amir , H. Dong , K. Tanaka , and M. J. Czaja . 2013. “Aging Promotes the Development of Diet‐Induced Murine Steatohepatitis but Not Steatosis.” Hepatology 57: 995–1004. 10.1002/hep.26099.. 23081825 PMC3566282

[acel70059-bib-0029] Ganta, V. C. , M. Choi , C. R. Farber , and B. H. Annex . 2019. “Antiangiogenic VEGF165b Regulates Macrophage Polarization via S100A8/S100A9 in Peripheral Artery Disease.” Circulation 139: 226–242. 10.1161/CIRCULATIONAHA.118.034165.. 30586702 PMC6322929

[acel70059-bib-0030] Ganta, V. C. , M. Choi , A. Kutateladze , and B. H. Annex . 2017. “VEGF165b Modulates Endothelial VEGFR1‐STAT3 Signaling Pathway and Angiogenesis in Human and Experimental Peripheral Arterial Disease.” Circulation Research 120: 282–295. 10.1161/CIRCRESAHA.116.309516.. 27974423 PMC5453503

[acel70059-bib-0031] Golledge, J. 2022. “Update on the Pathophysiology and Medical Treatment of Peripheral Artery Disease.” Nature Reviews. Cardiology 19: 456–474. 10.1038/s41569-021-00663-9.. 34997200

[acel70059-bib-0032] Gomez, C. R. , S. Hirano , B. T. Cutro , et al. 2007. “Advanced Age Exacerbates the Pulmonary Inflammatory Response After Lipopolysaccharide Exposure.” Critical Care Medicine 35: 246–251. 10.1097/01.CCM.0000251639.05135.E0.. 17133178

[acel70059-bib-0033] Gornik, H. L. , H. D. Aronow , P. P. Goodney , et al. 2024. “2024 ACC/AHA/AACVPR/APMA/ABC/SCAI/SVM/SVN/SVS/SIR/VESS Guideline for the Management of Lower Extremity Peripheral Artery Disease: A Report of the American College of Cardiology/American Heart Association Joint Committee on Clinical Practice Guidelines.” Circulation 149: e1313–e1410. 10.1161/CIR.0000000000001251.. 38743805 PMC12782132

[acel70059-bib-0034] Greiwe, J. S. , B. Cheng , D. C. Rubin , K. E. Yarasheski , and C. F. Semenkovich . 2001. “Resistance Exercise Decreases Skeletal Muscle Tumor Necrosis Factor Alpha in Frail Elderly Humans.” FASEB Journal 15: 475–482. 10.1096/fj.00-0274com.. 11156963

[acel70059-bib-0035] Grunewald, M. , S. Kumar , H. Sharife , et al. 2021. “Counteracting Age‐Related VEGF Signaling Insufficiency Promotes Healthy Aging and Extends Life Span.” Science 373: eabc8479. 10.1126/science.abc8479.. 34326210

[acel70059-bib-0036] He, H. , J. Xu , C. M. Warren , et al. 2012. “Endothelial Cells Provide an Instructive Niche for the Differentiation and Functional Polarization of M2‐Like Macrophages.” Blood 120: 3152–3162. 10.1182/blood-2012-04-422758.. 22919031 PMC3471522

[acel70059-bib-0037] Heil, M. , T. Ziegelhoeffer , S. Wagner , et al. 2004. “Collateral Artery Growth (Arteriogenesis) After Experimental Arterial Occlusion Is Impaired in Mice Lacking CC‐Chemokine Receptor‐2.” Circulation Research 94: 671–677. 10.1161/01.RES.0000122041.73808.B5.. 14963007

[acel70059-bib-0038] Kikuchi, R. , K. Nakamura , S. MacLauchlan , et al. 2014. “An Anti‐Angiogenic Isoform of VEGF‐A Contributes to Impaired Vascularization in Peripheral Artery Disease.” Nature Medicine 20: 1464–1471. 10.1038/nm.3703.. PMC4257756 25362254

[acel70059-bib-0039] Kim, M. S. , J. Hwang , D. K. Yon , et al. 2023. “Global Burden of Peripheral Artery Disease and Its Risk Factors, 1990–2019: A Systematic Analysis for the Global Burden of Disease Study 2019.” Lancet Global Health 11, no. 10: e1553–e1565. 10.1016/S2214-109X(23)00355-8. 37734799 PMC10522777

[acel70059-bib-0040] Kole, R. , A. R. Krainer , and S. Altman . 2012. “RNA Therapeutics: Beyond RNA Interference and Antisense Oligonucleotides.” Nature Reviews. Drug Discovery 11: 125–140. 10.1038/nrd3625.. 22262036 PMC4743652

[acel70059-bib-0041] Krasniewski, L. K. , P. Chakraborty , C.‐Y. Cui , et al. 2022. “Single‐Cell Analysis of Skeletal Muscle Macrophages Reveals Age‐Associated Functional Subpopulations.” eLife 11: e77974. 10.7554/eLife.77974. 36259488 PMC9629833

[acel70059-bib-0042] Krishnasamy, K. , A. Limbourg , T. Kapanadze , et al. 2017. “Blood Vessel Control of Macrophage Maturation Promotes Arteriogenesis in Ischemia.” Nature Communications 8: 952. 10.1038/s41467-017-00953-2. PMC5643305 29038527

[acel70059-bib-0043] Lähteenvuo, J. , and A. Rosenzweig . 2012. “Effects of Aging on Angiogenesis.” Circulation Research 110: 1252–1264. 10.1161/CIRCRESAHA.111.246116.. 22539758 PMC4101916

[acel70059-bib-0044] Larsen, M. , C. Bayard , H. Lepetitcorps , et al. 2017. “Elevated Neopterin Levels Predict Early Death in Older Hip‐Fracture Patients.” eBioMedicine 26: 157–164. 10.1016/j.ebiom.2017.11.003.. 29157836 PMC5832560

[acel70059-bib-0045] Lazarov, T. , S. Juarez‐Carreño , N. Cox , and F. Geissmann . 2023. “Physiology and Diseases of Tissue‐Resident Macrophages.” Nature 618: 698–707. 10.1038/s41586-023-06002-x.. 37344646 PMC10649266

[acel70059-bib-0046] Lee, B. P. , L. C. Pilling , F. Emond , et al. 2016. “Changes in the Expression of Splicing Factor Transcripts and Variations in Alternative Splicing Are Associated With Lifespan in Mice and Humans.” Aging Cell 15: 903–913. 10.1111/acel.12499.. 27363602 PMC5013025

[acel70059-bib-0047] Leosco, D. , G. Rengo , G. Iaccarino , et al. 2007. “Prior Exercise Improves Age‐Dependent Vascular Endothelial Growth Factor Downregulation and Angiogenesis Responses to Hind‐Limb Ischemia in Old Rats.” Journals of Gerontology Series A: Biological Sciences and Medical Sciences 62: 471–480. 10.1093/gerona/62.5.471.. 17522350

[acel70059-bib-0048] Li, C.‐J. , Y. Xiao , Y.‐C. Sun , et al. 2021. “Senescent Immune Cells Release Grancalcin to Promote Skeletal Aging.” Cell Metabolism 33: 1957–1973.e6. 10.1016/j.cmet.2021.08.009.. 34614408

[acel70059-bib-0049] Li, H. , Z. Wang , T. Ma , G. Wei , and T. Ni . 2017. “Alternative Splicing in Aging and Age‐Related Diseases.” Translational Medicine of Aging 1: 32–40. 10.1016/j.tma.2017.09.005..

[acel70059-bib-0050] Limbourg, A. , T. Korff , L. C. Napp , W. Schaper , H. Drexler , and F. P. Limbourg . 2009. “Evaluation of Postnatal Arteriogenesis and Angiogenesis in a Mouse Model of Hind‐Limb Ischemia.” Nature Protocols 4: 1737–1746. 10.1038/nprot.2009.185.. 19893509

[acel70059-bib-0051] Lin, C. L. , L. A. Bristol , L. Jin , et al. 1998. “Aberrant RNA Processing in a Neurodegenerative Disease: The Cause for Absent EAAT2, a Glutamate Transporter, in Amyotrophic Lateral Sclerosis.” Neuron 20: 589–602. 10.1016/s0896-6273(00)80997-6.. 9539131

[acel70059-bib-0052] Lin, J. B. , A. Sene , A. Santeford , et al. 2018. “Oxysterol Signatures Distinguish Age‐Related Macular Degeneration From Physiologic Aging.” eBioMedicine 32: 9–20. 10.1016/j.ebiom.2018.05.035.. 29903570 PMC6021272

[acel70059-bib-0053] Lumeng, C. N. , J. Liu , L. Geletka , et al. 2011. “Aging Is Associated With an Increase in T Cells and Inflammatory Macrophages in Visceral Adipose Tissue.” Journal of Immunology 187: 6208–6216. 10.4049/jimmunol.1102188.. PMC3237772 22075699

[acel70059-bib-0054] Mantovani, A. , S. Sozzani , M. Locati , P. Allavena , and A. Sica . 2002. “Macrophage Polarization: Tumor‐Associated Macrophages as a Paradigm for Polarized M2 Mononuclear Phagocytes.” Trends in Immunology 23: 549–555. 10.1016/s1471-4906(02)02302-5.. 12401408

[acel70059-bib-0055] Martini, H. , J. S. Iacovoni , D. Maggiorani , et al. 2019. “Aging Induces Cardiac Mesenchymal Stromal Cell Senescence and Promotes Endothelial Cell Fate of the CD90+ Subset.” Aging Cell 18: e13015. 10.1111/acel.13015.. 31353772 PMC6718537

[acel70059-bib-0056] Minhas, P. S. , L. Liu , P. K. Moon , et al. 2019. “Macrophage De Novo NAD+ Synthesis Specifies Immune Function in Aging and Inflammation.” Nature Immunology 20: 50–63. 10.1038/s41590-018-0255-3.. 30478397 PMC6768398

[acel70059-bib-0057] Morrison, A. , and A. W. Aday . 2022. “Sex as a Key Determinant of Peripheral Artery Disease: Epidemiology, Differential Outcomes, and Proposed Biological Mechanisms.” Canadian Journal of Cardiology 38: 601–611. 10.1016/j.cjca.2022.02.021.. 35231552 PMC9090953

[acel70059-bib-0058] Morrison, A. R. , T. O. Yarovinsky , B. D. Young , et al. 2014. “Chemokine‐Coupled β2 Integrin‐Induced Macrophage Rac2‐Myosin IIA Interaction Regulates VEGF‐A mRNA Stability and Arteriogenesis.” Journal of Experimental Medicine 211: 1957–1968. 10.1084/jem.20132130.. 25180062 PMC4172219

[acel70059-bib-0059] Moss, C. E. , S. A. Johnston , J. V. Kimble , et al. 2024. “Aging‐Related Defects in Macrophage Function Are Driven by MYC and USF1 Transcriptional Programs.” Cell Reports 43: 114073. 10.1016/j.celrep.2024.114073.. 38578825

[acel70059-bib-0060] Natrajan, M. S. , A. G. de la Fuente , A. H. Crawford , et al. 2015. “Retinoid X Receptor Activation Reverses Age‐Related Deficiencies in Myelin Debris Phagocytosis and Remyelination.” Brain 138: 3581–3597. 10.1093/brain/awv289.. 26463675 PMC4668920

[acel70059-bib-0061] Pabon, M. , S. Cheng , S. E. Altin , et al. 2022. “Sex Differences in Peripheral Artery Disease.” Circulation Research 130: 496–511. 10.1161/CIRCRESAHA.121.320702.. 35175843 PMC8919803

[acel70059-bib-0062] Pérez‐Gutiérrez, L. , and N. Ferrara . 2023. “Biology and Therapeutic Targeting of Vascular Endothelial Growth Factor A.” Nature Reviews. Molecular Cell Biology 24: 816–834. 10.1038/s41580-023-00631-w.. 37491579

[acel70059-bib-0063] Potente, M. , H. Gerhardt , and P. Carmeliet . 2011. “Basic and Therapeutic Aspects of Angiogenesis.” Cell 146: 873–887. 10.1016/j.cell.2011.08.039.. 21925313

[acel70059-bib-0064] Rawji, K. S. , A. M. H. Young , T. Ghosh , et al. 2020. “Niacin‐Mediated Rejuvenation of Macrophage/Microglia Enhances Remyelination of the Aging Central Nervous System.” Acta Neuropathologica 139: 893–909. 10.1007/s00401-020-02129-7.. 32030468 PMC7181452

[acel70059-bib-0065] Ryan, N. A. , K. A. Zwetsloot , L. M. Westerkamp , R. C. Hickner , W. E. Pofahl , and T. P. Gavin . 2006. “Lower Skeletal Muscle Capillarization and VEGF Expression in Aged vs. Young Men.” Journal of Applied Physiology (Bethesda, MD: 1985) 100: 178–185. 10.1152/japplphysiol.00827.2005.. 16166239

[acel70059-bib-0066] Shavlakadze, T. , J. McGeachie , and M. D. Grounds . 2010. “Delayed but Excellent Myogenic Stem Cell Response of Regenerating Geriatric Skeletal Muscles in Mice.” Biogerontology 11: 363–376. 10.1007/s10522-009-9260-0.. 20033288

[acel70059-bib-0067] Shenoy, P. , and A. Harugeri . 2015. “Elderly Patients' Participation in Clinical Trials.” Perspectives in Clinical Research 6: 184–189. 10.4103/2229-3485.167099.. 26623388 PMC4640010

[acel70059-bib-0068] Shireman, P. K. 2007. “The Chemokine System in Arteriogenesis and Hind Limb Ischemia.” Journal of Vascular Surgery 45, no. Suppl A: A48–A56. 10.1016/j.jvs.2007.02.030.. 17544024 PMC2680944

[acel70059-bib-0069] Simons, M. 2008. “Chapter 14 Assessment of Arteriogenesis.” In Methods in Enzymology, 331–342. Elsevier. 10.1016/S0076-6879(08)03014-0.. 19022066

[acel70059-bib-0070] Spiller, K. L. , R. R. Anfang , K. J. Spiller , et al. 2014. “The Role of Macrophage Phenotype in Vascularization of Tissue Engineering Scaffolds.” Biomaterials 35: 4477–4488. 10.1016/j.biomaterials.2014.02.012.. 24589361 PMC4000280

[acel70059-bib-0071] Takeda, Y. , S. Costa , E. Delamarre , et al. 2011. “Macrophage Skewing by Phd2 Haplodeficiency Prevents Ischaemia by Inducing Arteriogenesis.” Nature 479: 122–126. 10.1038/nature10507.. 21983962 PMC4659699

[acel70059-bib-0072] Tang, Y. , I. Horikawa , M. Ajiro , et al. 2013. “Downregulation of Splicing Factor SRSF3 Induces p53β, an Alternatively Spliced Isoform of p53 That Promotes Cellular Senescence.” Oncogene 32: 2792–2798. 10.1038/onc.2012.288.. 22777358 PMC6503963

[acel70059-bib-0073] Wagatsuma, A. 2006. “Effect of Aging on Expression of Angiogenesis‐Related Factors in Mouse Skeletal Muscle.” Experimental Gerontology 41: 49–54. 10.1016/j.exger.2005.10.003.. 16289925

[acel70059-bib-0074] Wang, L. , W. Hong , H. Zhu , et al. 2024. “Macrophage Senescence in Health and Diseases.” Acta Pharmaceutica Sinica B 14: 1508–1524. 10.1016/j.apsb.2024.01.008.. 38572110 PMC10985037

[acel70059-bib-0075] Wang, Y. , M. Wehling‐Henricks , G. Samengo , and J. G. Tidball . 2015. “Increases of M2a Macrophages and Fibrosis in Aging Muscle Are Influenced by Bone Marrow Aging and Negatively Regulated by Muscle‐Derived Nitric Oxide.” Aging Cell 14: 678–688. 10.1111/acel.12350.. 26009878 PMC4531081

[acel70059-bib-0076] Wang, Y. , S. S. Welc , M. Wehling‐Henricks , and J. G. Tidball . 2018. “Myeloid Cell‐Derived Tumor Necrosis Factor‐Alpha Promotes Sarcopenia and Regulates Muscle Cell Fusion With Aging Muscle Fibers.” Aging Cell 17: e12828. 10.1111/acel.12828.. 30256507 PMC6260911

[acel70059-bib-0077] Willenborg, S. , T. Lucas , G. van Loo , et al. 2012. “CCR2 Recruits an Inflammatory Macrophage Subpopulation Critical for Angiogenesis in Tissue Repair.” Blood 120: 613–625. 10.1182/blood-2012-01-403386.. 22577176

[acel70059-bib-0078] Woolard, J. , W.‐Y. Wang , H. S. Bevan , et al. 2004. “VEGF165b, an Inhibitory Vascular Endothelial Growth Factor Splice Variant: Mechanism of Action, In Vivo Effect on Angiogenesis and Endogenous Protein Expression.” Cancer Research 64: 7822–7835. 10.1158/0008-5472.CAN-04-0934.. 15520188

[acel70059-bib-0079] Wynn, T. A. , and K. M. Vannella . 2016. “Macrophages in Tissue Repair, Regeneration, and Fibrosis.” Immunity 44: 450–462. 10.1016/j.immuni.2016.02.015.. 26982353 PMC4794754

[acel70059-bib-0080] Yin, H. , F. Price , and M. A. Rudnicki . 2013. “Satellite Cells and the Muscle Stem Cell Niche.” Physiological Reviews 93: 23–67. 10.1152/physrev.00043.2011.. 23303905 PMC4073943

[acel70059-bib-0081] Zhang, J. , J. Muri , G. Fitzgerald , et al. 2020. “Endothelial Lactate Controls Muscle Regeneration From Ischemia by Inducing M2‐Like Macrophage Polarization.” Cell Metabolism 31: 1136–1153.e7. 10.1016/j.cmet.2020.05.004.. 32492393 PMC7267778

