# Supplementary material for: Senescent Macrophages Promote Age‐Related Revascularization Impairment by Increasing Antiangiogenic VEGF‐A165B Expression
Source: Aging Cell. 2025 Apr 17;24(7):e70059. doi: 10.1111/acel.70059 (PMC12266784; doi:10.1111/acel.70059)
Supplement: Supplementary file 1 — Figure S1. Aging promotes the macrophages senescence in hindlimb skeletal muscle. Gating strategy of macrophages in the hindlimb skeletal muscle. Representative flow cytometry plots for macrophages of hindlimb skeletal muscle stained with CD86, blank control and fluorescence minus one (FMO) control. Representative flow cytometry plots for macrophages of hindlimb skeletal muscle stained with CD206, blank control and FMO control. (d, g) Representative flow cytometry plots (d) and quantification (g) of the percent of SPiDER‐βGal+F4/80+ cells in the hindlimb skeletal muscle (n = 6). (e, h) Representative flow cytometry plots (e) and quantification (h) of the percent of SPiDER‐βGal+CD86+ cells in the hindlimb skeletal muscle (n = 6). (f, i) Representative flow cytometry plots (f) and quantification (i) of the percent of SPiDER‐βGal+CD206+ cells in the hindlimb skeletal muscle (n = 6). Representative flow cytometry plots for macrophages of hindlimb skeletal muscle stained with SPiDER‐βGal, blank control and FMO control. Immunoblot images and quantification for MHCII or LYVE1 protein levels in non‐SMPs and SMPs isolated from hindlimb skeletal muscle of 24‐month‐old mice (n = 3). (l, m) Real‐time PCR analysis of Mhcii (l) and Lyve1 (m) in non‐SMPs and SMPs isolated from hindlimb skeletal muscle of 24‐month‐old mice (n = 3). Unpaired t‐tests. Error bars represent SEM. **, ***, **** and ns denote p < 0.01, p < 0.001, and p < 0.0001, not significant, respectively. Figure S2. Senescent macrophages actively affect proliferation and eNOS phosphorylation of skeletal muscle ECs in vivo. (a, b) Representative CD31 (green), aSMA (red), and KI67 (gray) immunofluorescent images (a) and quantification (b) on gastrocnemius cross sections of mice transferred with SMPs or non‐SMPs at 7 days after HLI (n = 6; scale bar = 50 μm). (c, d) Representative CD31 (green) and KI67 (red) immunofluorescent images (c) and quantification (d) on gastrocnemius cross sections of mice transferred with SMPs [file ACEL-24-e70059-s001.zip › acel70059-sup-0001-FigureS1-S7/acel70059-sup-0001-FigureS1-S7/acel70059-sup-0001-FigureS1-S7/Revised Supplementary figure legends.docx]

**FIGURE** **S1 Aging promotes the macrophages senescence in hindlimb skeletal muscle**.
(a) Gating strategy of macrophages in the hindlimb skeletal muscle.
(b) Representative flow cytometry plots for macrophages of hindlimb skeletal muscle stained with CD86, blank control and fluorescence minus one (FMO) control.
(c) Representative flow cytometry plots for macrophages of hindlimb skeletal muscle stained with CD206, blank control and FMO control.
(d, g) Representative flow cytometry plots (d) and quantification (g) of the percent of SPiDER-βGal^+^F4/80^+^ cells in the hindlimb skeletal muscle (*n* = 6).
(e, h) Representative flow cytometry plots (e) and quantification (h) of the percent of SPiDER-βGal^+^CD86^+^ cells in the hindlimb skeletal muscle (*n* = 6).
(f, i) Representative flow cytometry plots (f) and quantification (i) of the percent of SPiDER-βGal^+^CD206^+^ cells in the hindlimb skeletal muscle (*n* = 6).
(j) Representative flow cytometry plots for macrophages of hindlimb skeletal muscle stained with SPiDER-βGal, blank control and FMO control.
(k) Immunoblot images and quantification for MHCII or LYVE1 protein levels in non-SMPs and SMPs isolated from hindlimb skeletal muscle of 24-month-old mice (*n* = 3).
(l, m) Real-time qPCR analysis of *Mhcii* (l) and *Lyve1* (m) in non-SMPs and SMPs isolated from hindlimb skeletal muscle of 24-month-old mice (*n* = 3).
Unpaired *t*-tests. Error bars represent SEM. **, ***, **** and ns denote *p* < 0.01, 0.001, and 0.0001, not significant, respectively.

**Figure** **S2 Senescent macrophages actively affect proliferation and eNOS phosphorylation of skeletal muscle ECs in vivo.**
(a, b) Representative CD31 (green), α-SMA (red), and KI67 (gray) immunofluorescent images (a) and quantification (b) on gastrocnemius cross sections of mice transferred with SMPs or non-SMPs at 7 days after HLI (*n* = 6; scale bar = 50 μm).
(c, d) Representative CD31 (green) and KI67 (red) immunofluorescent images (c) and quantification (d) on gastrocnemius cross sections of mice transferred with SMPs or non-SMPs at 7 days after HLI (*n* = 6; scale bar = 20 μm).
(e, f) Representative p-eNOS immunohistochemical images (e) and quantification (f) on gastrocnemius cross sections of mice transferred with SMPs or non-SMPs at 7 days after HLI (*n* = 6; scale bar = 100 μm).
(g) NO levels in ischemic hindlimb muscle ECs of mice transferred with SMPs or non-SMPs at 7 days (*n* = 3).
One-way ANOVA. Error bars represent SEM. ***, **** and ns denote *p* < 0.001, 0.0001 and not significant, respectively.

**FIGURE** **S3 Features of senescent macrophages in vitro are replicated in vivo.**
(a) Scheme showing the experiment of senescent BMDM model.
(b) Representative F4/80 immunofluorescent images of YBMDMs and SBMDMs (*n* = 3; scale bar = 20 μm).
(c, d) Representative flow cytometry plots (c) and quantification (d) of the percent of F4/80^+^ cells (*n* = 3).
(e, f) Representative images (e) and quantification (f) of SA-β-gal staining positive macrophages (*n* = 3; scale bar = 75 μm).
(g) Immunoblot images and quantification for P21 or LMNB1 protein levels in YBMDMs and SBMDMs (*n* = 3).
(h) Immunoblot images and quantification for MHCII or LYVE1 protein levels in YBMDMs and SBMDMs (*n* = 3).
(i, j) Representative images of KI67 (red) and DAPI (blue) immunostaining (i) and quantification (j) of KI67^+^ cells in YBMDMs and SBMDMs (*n* = 3; scale bar = 20 μm).
(k, l) Representative images of γ-H2AX (red) and DAPI (blue) immunostaining (k) and quantification (l) of γ-H2AX ^+^ cells in YBMDMs and SBMDMs (*n* = 3; scale bar = 20 μm).
(m) The heatmap of real-time qPCR analysis for SASP markers, senescent markers, and Sirt family in YBMDMs and SBMDMs (*n* = 3).
(n-s) Real-time qPCR analysis of SASP markers (n), senescent markers (o), Sirt family (p), pro- or anti-inflammatory markers (q), *Mhcii* (r) and *Lyve1* (s) in YBMDMs and SBMDMs (*n* = 3)
Real-time qPCR experiment of pro- or anti-inflammatory markers was analyzed by one-way ANOVA, others were analyzed by Unpaired *t*-tests. Error bars represent SEM. *, **, ***, **** denote *p* < 0.05, 0.01, 0.001, and 0.0001, respectively.

**FIGURE** **S4 DAF-FM DA staining control and Flt1 mRNA levels in mECs**.
(a) Representative flow cytometry plots for mECs with blank control and DAF-FM DA.
(b) Quantification of the percent of NO^low^ mECs treated with Y-CM, S-CM or control media (*n* = 3).
(c) Real-time qPCR analysis of *Flt1* in mECs treated with Y-CM, S-CM and control media (*n* = 3).
One-way ANOVA. Error bars represent SEM. * and ns denote *p* < 0.05 and not significant, respectively.

**FIGURE** **S5 Senescent macrophages highly express VEGF-A165B in vivo and in vitro**.
(a-c) Real-time qPCR analysis of *Vegf-a* (a), *Vegf-a165a* (b) and *Vegf-a165b* (c) expression in young and senescent BMDMs (*n* = 3).
(d-f) Real-time qPCR analysis of *Vegfa* (d), *Vegf-a165a* (e) and *Vegf-a165b* (f) expression in non-SMPs and SMPS of ischemic hindlimb muscle (*n* = 3).
(g-i) ELISA analysis of VEGF-A (g), VEGF-A165A (h) and VEGF-A165B (i) protein levels in CM from young and senescent BMDMs (*n* = 6).
Unpaired *t*-tests. Error bars represent SEM. *, ** and ns denote *p* < 0.05, 0.01, and not significant, respectively.

**FIGURE** **S6 Knockdown efficiency of VEGF-A165B in senescent BMDMs**.
(a) Immunoblot images and quantification for VEGF-A165B protein levels in SBMDMs treated with *Vegf-a165b siRNA* or control siRNA (*n* = 3).
(b) Real-time qPCR analysis of *Vegf-a165b* expression in SBMDMs treated with *Vegf-a165b siRNA* or control siRNA (*n* = 3).
(c) Quantification of the percent of NO^low^ mECs in S-CM treated with control siRNA or *Vegf-a165b siRNA* (*n* = 3).
(d) Quantification of the percent of NO^low^ mECs in S-CM treated with isotype IgG or VEGF-A165B antibody (*n* = 3).
Unpaired *t*-tests. Error bars represent SEM. ** denote *p* < 0.01.

**FIGURE** **S7 VEGF-A165B knockdown rescue the effect of senescent macrophages on proliferation and eNOS phosphorylation of skeletal muscle ECs in vivo**.
(a) Immunoblot images and quantification for VEGF-A165B protein levels in hindlimb skeletal muscle macrophages of mice injected with AAV-sh*Vegf-a165b* or AAV-Scramble (*n* = 3).
(b) Real-time qPCR analysis of *Vegf-a165b* expression in hindlimb skeletal muscle macrophages of mice injected with AAV-sh*Vegf-a165b* or AAV-Scramble (*n* = 3).
(c, d) Representative CD31 (green), α-SMA (red), and KI67 (gray) immunofluorescent images (c) and quantification (d) on gastrocnemius cross sections of mice transferred with SMPs or non-SMPs from mice injected with AAV-sh*Vegf-a165b* or AAV-Scramble at 7 days after HLI (*n* = 6; scale bar = 50 μm).
(e, f) Representative CD31 (green) and KI67 (red) immunofluorescent images (e) and quantification (f) on gastrocnemius cross sections of mice transferred with SMPs or non-SMPs from mice injected with AAV-sh*Vegf-a165b* or AAV-Scramble at 7 days after HLI (*n* = 6; scale bar = 20 μm).
(g, h) Representative p-eNOS immunohistochemical images (g) and quantification (h) on gastrocnemius cross sections of mice transferred with SMPs or non-SMPs from mice injected with AAV-sh*Vegf-a165b* or AAV-Scramble at 7 days after HLI (*n* = 6; scale bar = 100 μm).
(i) NO levels in ischemic hindlimb muscle ECs of mice transferred with SMPs or non-SMPs from mice injected with AAV-sh*Vegf-a165b* or AAV-Scramble at 7 days (*n* = 3).
Immunoblot and Real-time qPCR experiments of Vegf-a165b levels in hindlimb skeletal muscle macrophages was analyzed by unpaired *t*-tests, others were analyzed by one-way ANOVA. Error bars represent SEM. ***, **** and ns denote *p* < 0.001, 0.0001 and not significant, respectively.
