# Supplementary figures and images for: Senescent Macrophages Promote Age‐Related Revascularization Impairment by Increasing Antiangiogenic VEGF‐A165B Expression
Source: Aging Cell. 2025 Apr 17;24(7):e70059. doi: 10.1111/acel.70059 (PMC12266784; doi:10.1111/acel.70059)

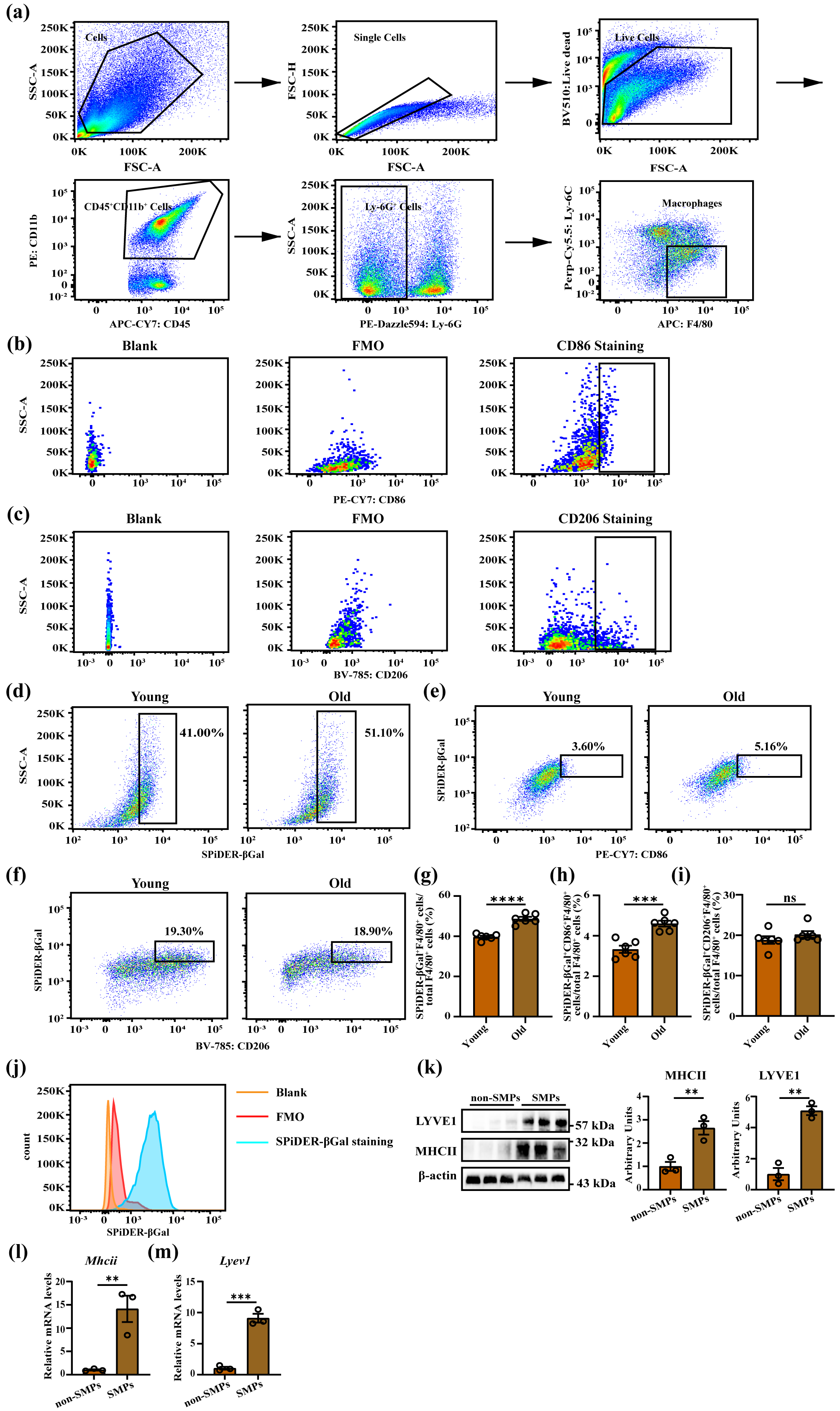

Supplement: Supplementary file 1 — Figure S1. Aging promotes the macrophages senescence in hindlimb skeletal muscle. Gating strategy of macrophages in the hindlimb skeletal muscle. Representative flow cytometry plots for macrophages of hindlimb skeletal muscle stained with CD86, blank control and fluorescence minus one (FMO) control. Representative flow cytometry plots for macrophages of hindlimb skeletal muscle stained with CD206, blank control and FMO control. (d, g) Representative flow cytometry plots (d) and quantification (g) of the percent of SPiDER‐βGal+F4/80+ cells in the hindlimb skeletal muscle (n = 6). (e, h) Representative flow cytometry plots (e) and quantification (h) of the percent of SPiDER‐βGal+CD86+ cells in the hindlimb skeletal muscle (n = 6). (f, i) Representative flow cytometry plots (f) and quantification (i) of the percent of SPiDER‐βGal+CD206+ cells in the hindlimb skeletal muscle (n = 6). Representative flow cytometry plots for macrophages of hindlimb skeletal muscle stained with SPiDER‐βGal, blank control and FMO control. Immunoblot images and quantification for MHCII or LYVE1 protein levels in non‐SMPs and SMPs isolated from hindlimb skeletal muscle of 24‐month‐old mice (n = 3). (l, m) Real‐time PCR analysis of Mhcii (l) and Lyve1 (m) in non‐SMPs and SMPs isolated from hindlimb skeletal muscle of 24‐month‐old mice (n = 3). Unpaired t‐tests. Error bars represent SEM. **, ***, **** and ns denote p < 0.01, p < 0.001, and p < 0.0001, not significant, respectively. Figure S2. Senescent macrophages actively affect proliferation and eNOS phosphorylation of skeletal muscle ECs in vivo. (a, b) Representative CD31 (green), aSMA (red), and KI67 (gray) immunofluorescent images (a) and quantification (b) on gastrocnemius cross sections of mice transferred with SMPs or non‐SMPs at 7 days after HLI (n = 6; scale bar = 50 μm). (c, d) Representative CD31 (green) and KI67 (red) immunofluorescent images (c) and quantification (d) on gastrocnemius cross sections of mice transferred with SMPs [file ACEL-24-e70059-s001.zip › acel70059-sup-0001-FigureS1-S7/acel70059-sup-0001-FigureS1-S7/acel70059-sup-0001-FigureS1-S7/acel70059-sup-0001-FigureS1.tif]

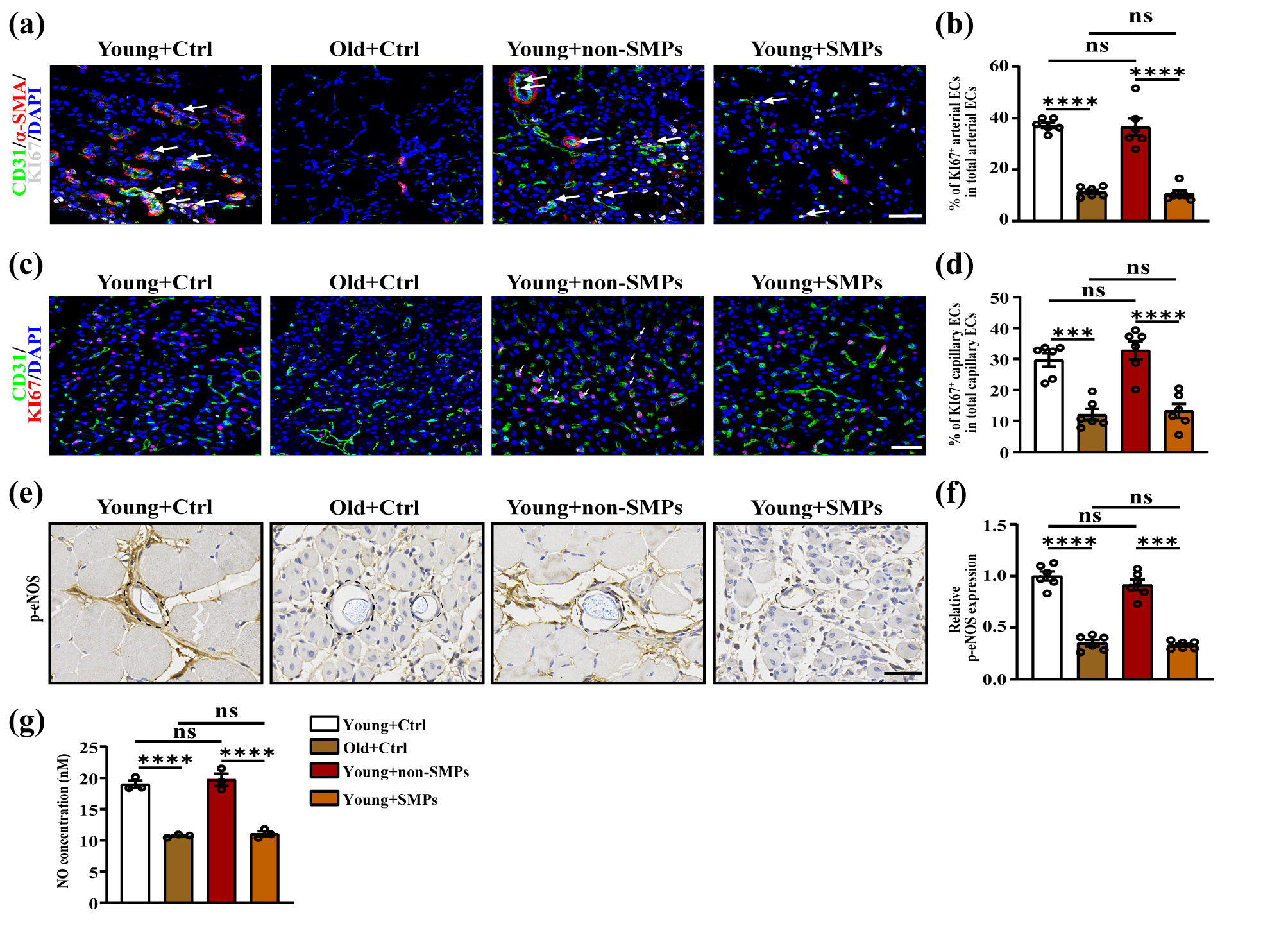

Supplement: Supplementary file 1 — Figure S1. Aging promotes the macrophages senescence in hindlimb skeletal muscle. Gating strategy of macrophages in the hindlimb skeletal muscle. Representative flow cytometry plots for macrophages of hindlimb skeletal muscle stained with CD86, blank control and fluorescence minus one (FMO) control. Representative flow cytometry plots for macrophages of hindlimb skeletal muscle stained with CD206, blank control and FMO control. (d, g) Representative flow cytometry plots (d) and quantification (g) of the percent of SPiDER‐βGal+F4/80+ cells in the hindlimb skeletal muscle (n = 6). (e, h) Representative flow cytometry plots (e) and quantification (h) of the percent of SPiDER‐βGal+CD86+ cells in the hindlimb skeletal muscle (n = 6). (f, i) Representative flow cytometry plots (f) and quantification (i) of the percent of SPiDER‐βGal+CD206+ cells in the hindlimb skeletal muscle (n = 6). Representative flow cytometry plots for macrophages of hindlimb skeletal muscle stained with SPiDER‐βGal, blank control and FMO control. Immunoblot images and quantification for MHCII or LYVE1 protein levels in non‐SMPs and SMPs isolated from hindlimb skeletal muscle of 24‐month‐old mice (n = 3). (l, m) Real‐time PCR analysis of Mhcii (l) and Lyve1 (m) in non‐SMPs and SMPs isolated from hindlimb skeletal muscle of 24‐month‐old mice (n = 3). Unpaired t‐tests. Error bars represent SEM. **, ***, **** and ns denote p < 0.01, p < 0.001, and p < 0.0001, not significant, respectively. Figure S2. Senescent macrophages actively affect proliferation and eNOS phosphorylation of skeletal muscle ECs in vivo. (a, b) Representative CD31 (green), aSMA (red), and KI67 (gray) immunofluorescent images (a) and quantification (b) on gastrocnemius cross sections of mice transferred with SMPs or non‐SMPs at 7 days after HLI (n = 6; scale bar = 50 μm). (c, d) Representative CD31 (green) and KI67 (red) immunofluorescent images (c) and quantification (d) on gastrocnemius cross sections of mice transferred with SMPs [file ACEL-24-e70059-s001.zip › acel70059-sup-0001-FigureS1-S7/acel70059-sup-0001-FigureS1-S7/acel70059-sup-0001-FigureS1-S7/acel70059-sup-0002-FigureS2.tif]

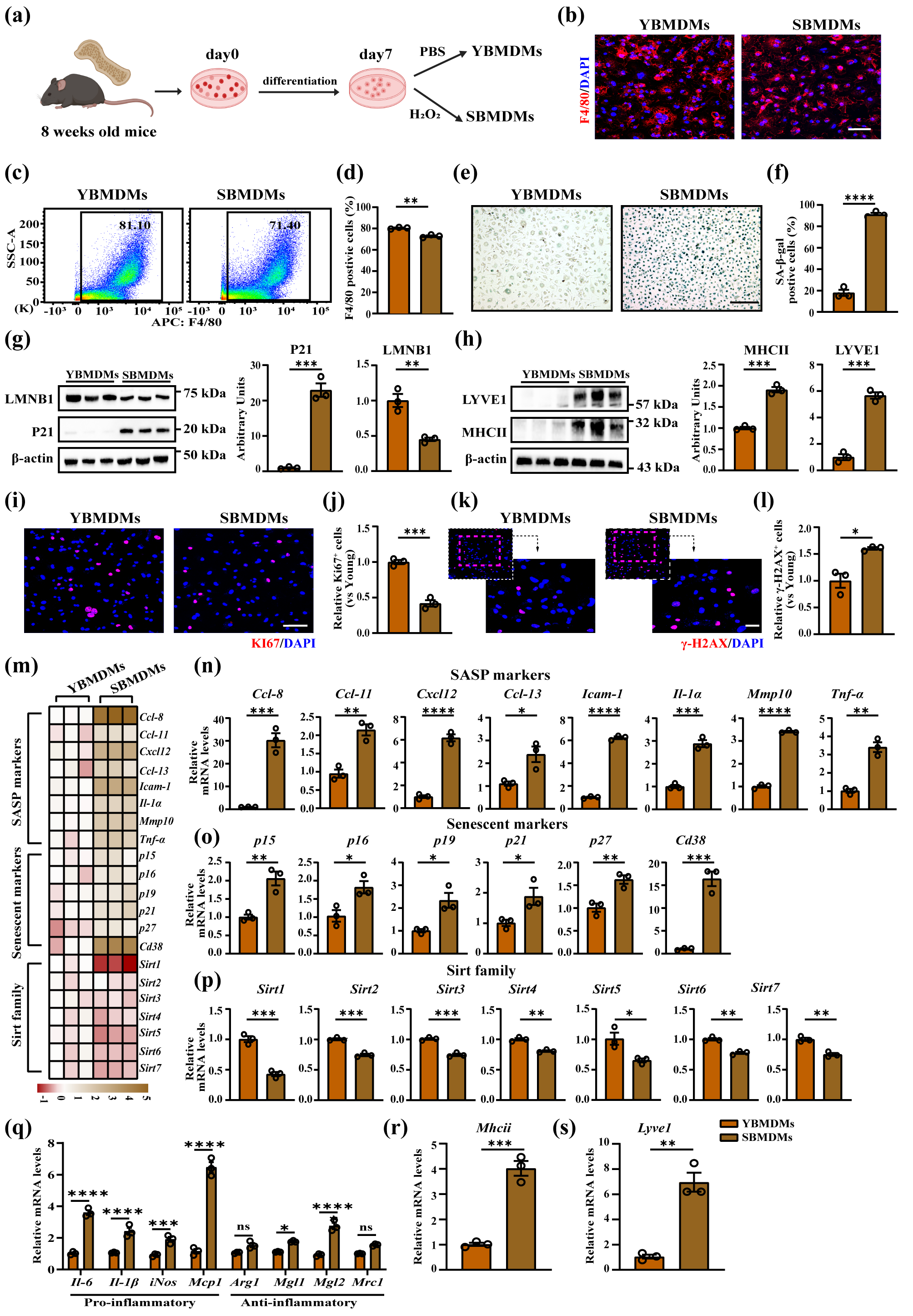

Supplement: Supplementary file 1 — Figure S1. Aging promotes the macrophages senescence in hindlimb skeletal muscle. Gating strategy of macrophages in the hindlimb skeletal muscle. Representative flow cytometry plots for macrophages of hindlimb skeletal muscle stained with CD86, blank control and fluorescence minus one (FMO) control. Representative flow cytometry plots for macrophages of hindlimb skeletal muscle stained with CD206, blank control and FMO control. (d, g) Representative flow cytometry plots (d) and quantification (g) of the percent of SPiDER‐βGal+F4/80+ cells in the hindlimb skeletal muscle (n = 6). (e, h) Representative flow cytometry plots (e) and quantification (h) of the percent of SPiDER‐βGal+CD86+ cells in the hindlimb skeletal muscle (n = 6). (f, i) Representative flow cytometry plots (f) and quantification (i) of the percent of SPiDER‐βGal+CD206+ cells in the hindlimb skeletal muscle (n = 6). Representative flow cytometry plots for macrophages of hindlimb skeletal muscle stained with SPiDER‐βGal, blank control and FMO control. Immunoblot images and quantification for MHCII or LYVE1 protein levels in non‐SMPs and SMPs isolated from hindlimb skeletal muscle of 24‐month‐old mice (n = 3). (l, m) Real‐time PCR analysis of Mhcii (l) and Lyve1 (m) in non‐SMPs and SMPs isolated from hindlimb skeletal muscle of 24‐month‐old mice (n = 3). Unpaired t‐tests. Error bars represent SEM. **, ***, **** and ns denote p < 0.01, p < 0.001, and p < 0.0001, not significant, respectively. Figure S2. Senescent macrophages actively affect proliferation and eNOS phosphorylation of skeletal muscle ECs in vivo. (a, b) Representative CD31 (green), aSMA (red), and KI67 (gray) immunofluorescent images (a) and quantification (b) on gastrocnemius cross sections of mice transferred with SMPs or non‐SMPs at 7 days after HLI (n = 6; scale bar = 50 μm). (c, d) Representative CD31 (green) and KI67 (red) immunofluorescent images (c) and quantification (d) on gastrocnemius cross sections of mice transferred with SMPs [file ACEL-24-e70059-s001.zip › acel70059-sup-0001-FigureS1-S7/acel70059-sup-0001-FigureS1-S7/acel70059-sup-0001-FigureS1-S7/acel70059-sup-0003-FigureS3.tif]

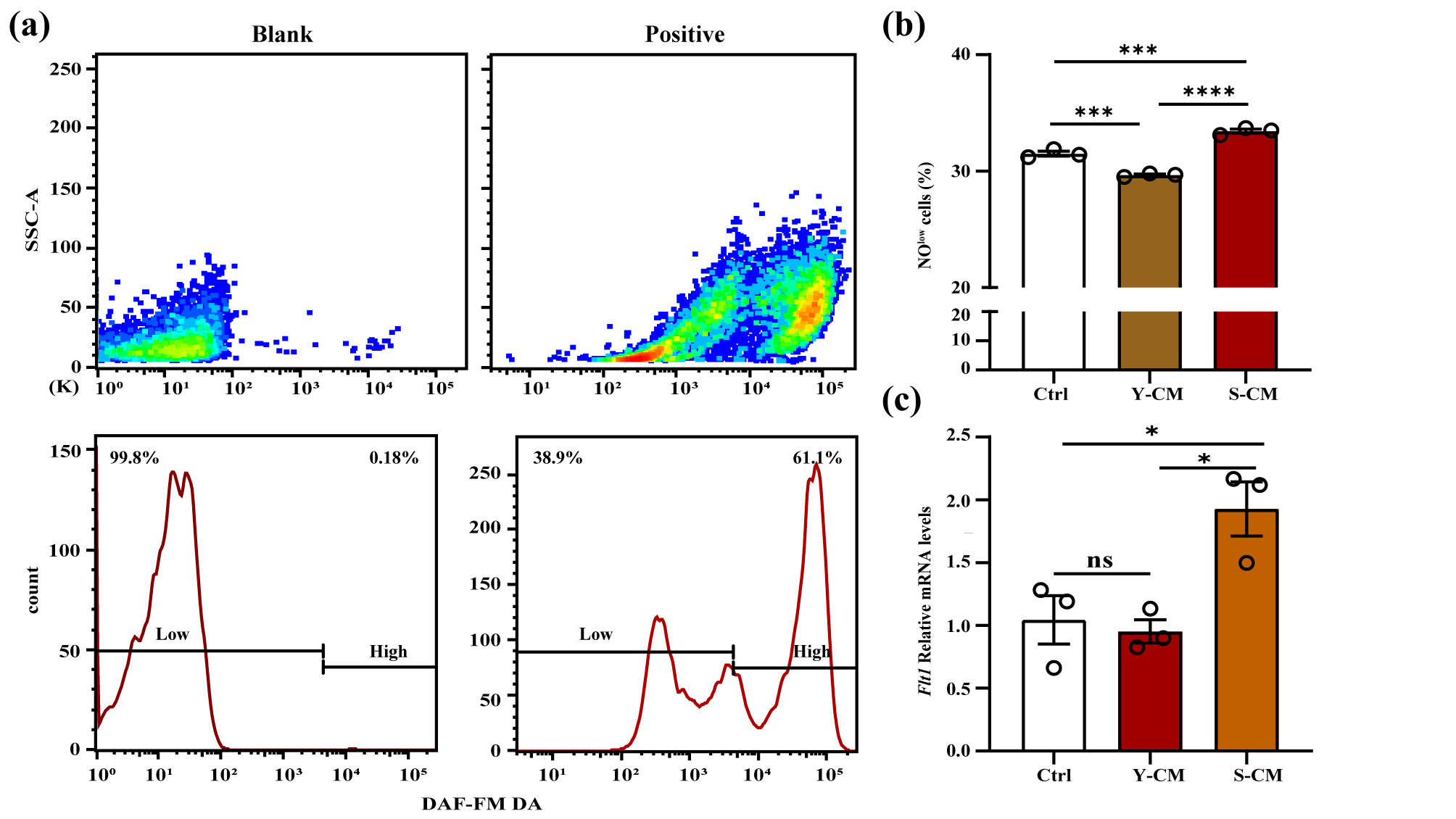

Supplement: Supplementary file 1 — Figure S1. Aging promotes the macrophages senescence in hindlimb skeletal muscle. Gating strategy of macrophages in the hindlimb skeletal muscle. Representative flow cytometry plots for macrophages of hindlimb skeletal muscle stained with CD86, blank control and fluorescence minus one (FMO) control. Representative flow cytometry plots for macrophages of hindlimb skeletal muscle stained with CD206, blank control and FMO control. (d, g) Representative flow cytometry plots (d) and quantification (g) of the percent of SPiDER‐βGal+F4/80+ cells in the hindlimb skeletal muscle (n = 6). (e, h) Representative flow cytometry plots (e) and quantification (h) of the percent of SPiDER‐βGal+CD86+ cells in the hindlimb skeletal muscle (n = 6). (f, i) Representative flow cytometry plots (f) and quantification (i) of the percent of SPiDER‐βGal+CD206+ cells in the hindlimb skeletal muscle (n = 6). Representative flow cytometry plots for macrophages of hindlimb skeletal muscle stained with SPiDER‐βGal, blank control and FMO control. Immunoblot images and quantification for MHCII or LYVE1 protein levels in non‐SMPs and SMPs isolated from hindlimb skeletal muscle of 24‐month‐old mice (n = 3). (l, m) Real‐time PCR analysis of Mhcii (l) and Lyve1 (m) in non‐SMPs and SMPs isolated from hindlimb skeletal muscle of 24‐month‐old mice (n = 3). Unpaired t‐tests. Error bars represent SEM. **, ***, **** and ns denote p < 0.01, p < 0.001, and p < 0.0001, not significant, respectively. Figure S2. Senescent macrophages actively affect proliferation and eNOS phosphorylation of skeletal muscle ECs in vivo. (a, b) Representative CD31 (green), aSMA (red), and KI67 (gray) immunofluorescent images (a) and quantification (b) on gastrocnemius cross sections of mice transferred with SMPs or non‐SMPs at 7 days after HLI (n = 6; scale bar = 50 μm). (c, d) Representative CD31 (green) and KI67 (red) immunofluorescent images (c) and quantification (d) on gastrocnemius cross sections of mice transferred with SMPs [file ACEL-24-e70059-s001.zip › acel70059-sup-0001-FigureS1-S7/acel70059-sup-0001-FigureS1-S7/acel70059-sup-0001-FigureS1-S7/acel70059-sup-0004-FigureS4.tif]

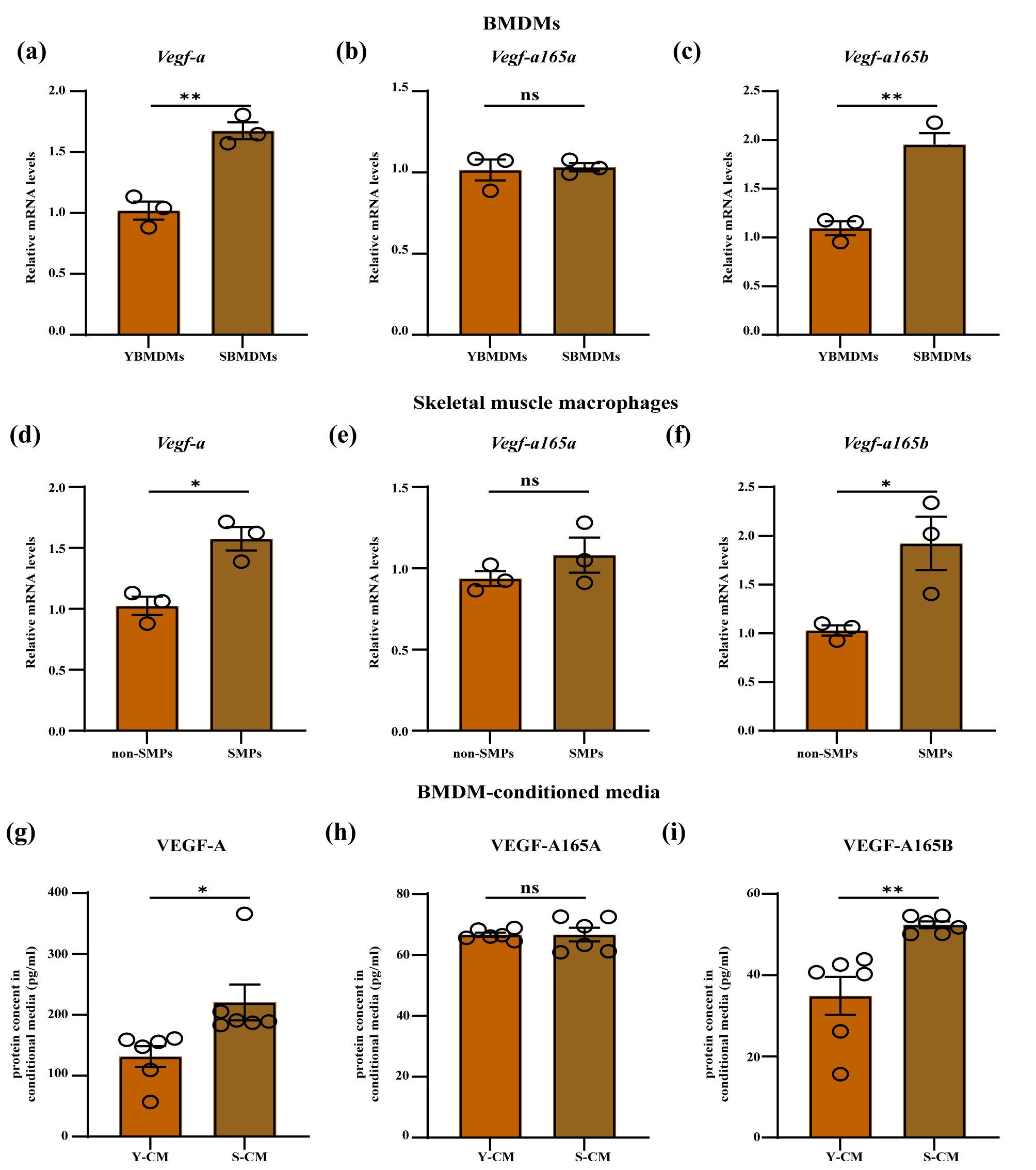

Supplement: Supplementary file 1 — Figure S1. Aging promotes the macrophages senescence in hindlimb skeletal muscle. Gating strategy of macrophages in the hindlimb skeletal muscle. Representative flow cytometry plots for macrophages of hindlimb skeletal muscle stained with CD86, blank control and fluorescence minus one (FMO) control. Representative flow cytometry plots for macrophages of hindlimb skeletal muscle stained with CD206, blank control and FMO control. (d, g) Representative flow cytometry plots (d) and quantification (g) of the percent of SPiDER‐βGal+F4/80+ cells in the hindlimb skeletal muscle (n = 6). (e, h) Representative flow cytometry plots (e) and quantification (h) of the percent of SPiDER‐βGal+CD86+ cells in the hindlimb skeletal muscle (n = 6). (f, i) Representative flow cytometry plots (f) and quantification (i) of the percent of SPiDER‐βGal+CD206+ cells in the hindlimb skeletal muscle (n = 6). Representative flow cytometry plots for macrophages of hindlimb skeletal muscle stained with SPiDER‐βGal, blank control and FMO control. Immunoblot images and quantification for MHCII or LYVE1 protein levels in non‐SMPs and SMPs isolated from hindlimb skeletal muscle of 24‐month‐old mice (n = 3). (l, m) Real‐time PCR analysis of Mhcii (l) and Lyve1 (m) in non‐SMPs and SMPs isolated from hindlimb skeletal muscle of 24‐month‐old mice (n = 3). Unpaired t‐tests. Error bars represent SEM. **, ***, **** and ns denote p < 0.01, p < 0.001, and p < 0.0001, not significant, respectively. Figure S2. Senescent macrophages actively affect proliferation and eNOS phosphorylation of skeletal muscle ECs in vivo. (a, b) Representative CD31 (green), aSMA (red), and KI67 (gray) immunofluorescent images (a) and quantification (b) on gastrocnemius cross sections of mice transferred with SMPs or non‐SMPs at 7 days after HLI (n = 6; scale bar = 50 μm). (c, d) Representative CD31 (green) and KI67 (red) immunofluorescent images (c) and quantification (d) on gastrocnemius cross sections of mice transferred with SMPs [file ACEL-24-e70059-s001.zip › acel70059-sup-0001-FigureS1-S7/acel70059-sup-0001-FigureS1-S7/acel70059-sup-0001-FigureS1-S7/acel70059-sup-0005-FigureS5.tif]

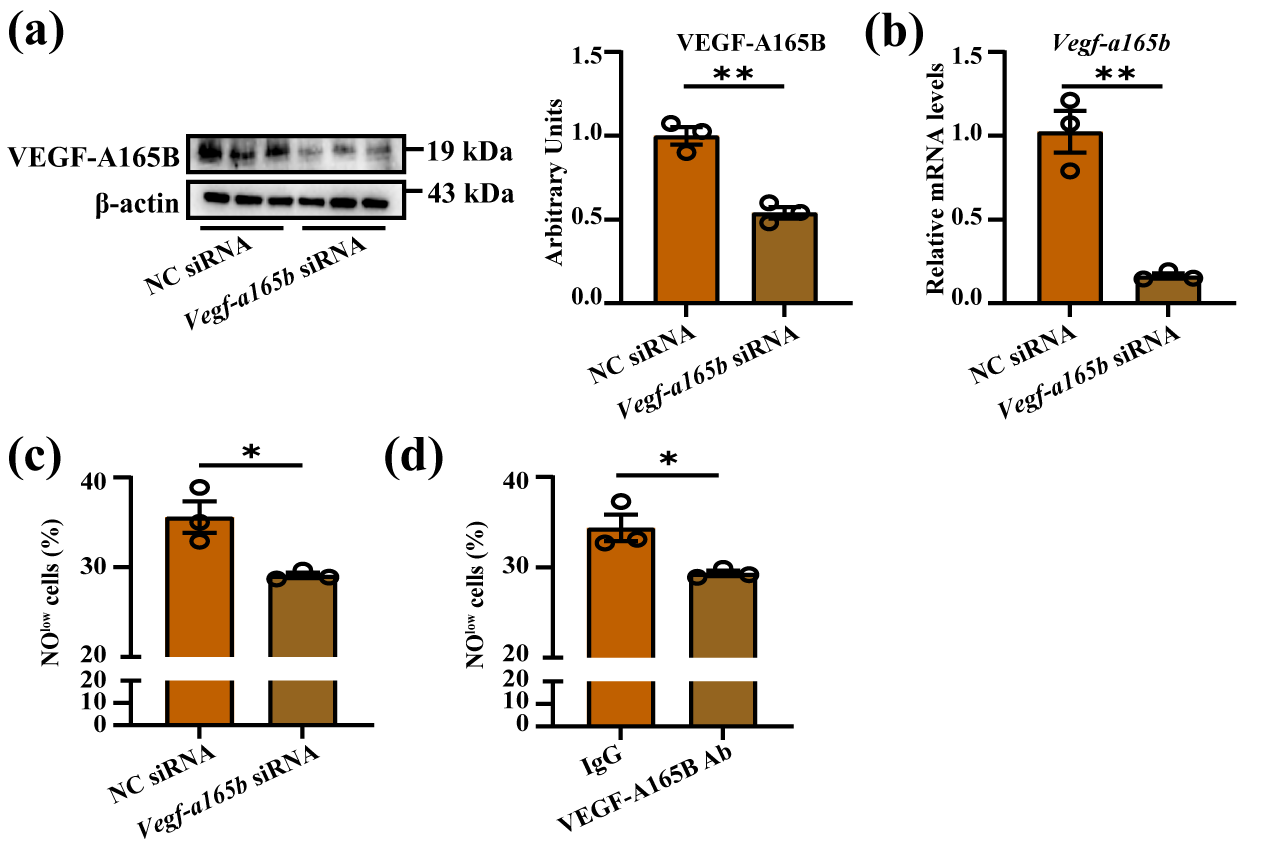

Supplement: Supplementary file 1 — Figure S1. Aging promotes the macrophages senescence in hindlimb skeletal muscle. Gating strategy of macrophages in the hindlimb skeletal muscle. Representative flow cytometry plots for macrophages of hindlimb skeletal muscle stained with CD86, blank control and fluorescence minus one (FMO) control. Representative flow cytometry plots for macrophages of hindlimb skeletal muscle stained with CD206, blank control and FMO control. (d, g) Representative flow cytometry plots (d) and quantification (g) of the percent of SPiDER‐βGal+F4/80+ cells in the hindlimb skeletal muscle (n = 6). (e, h) Representative flow cytometry plots (e) and quantification (h) of the percent of SPiDER‐βGal+CD86+ cells in the hindlimb skeletal muscle (n = 6). (f, i) Representative flow cytometry plots (f) and quantification (i) of the percent of SPiDER‐βGal+CD206+ cells in the hindlimb skeletal muscle (n = 6). Representative flow cytometry plots for macrophages of hindlimb skeletal muscle stained with SPiDER‐βGal, blank control and FMO control. Immunoblot images and quantification for MHCII or LYVE1 protein levels in non‐SMPs and SMPs isolated from hindlimb skeletal muscle of 24‐month‐old mice (n = 3). (l, m) Real‐time PCR analysis of Mhcii (l) and Lyve1 (m) in non‐SMPs and SMPs isolated from hindlimb skeletal muscle of 24‐month‐old mice (n = 3). Unpaired t‐tests. Error bars represent SEM. **, ***, **** and ns denote p < 0.01, p < 0.001, and p < 0.0001, not significant, respectively. Figure S2. Senescent macrophages actively affect proliferation and eNOS phosphorylation of skeletal muscle ECs in vivo. (a, b) Representative CD31 (green), aSMA (red), and KI67 (gray) immunofluorescent images (a) and quantification (b) on gastrocnemius cross sections of mice transferred with SMPs or non‐SMPs at 7 days after HLI (n = 6; scale bar = 50 μm). (c, d) Representative CD31 (green) and KI67 (red) immunofluorescent images (c) and quantification (d) on gastrocnemius cross sections of mice transferred with SMPs [file ACEL-24-e70059-s001.zip › acel70059-sup-0001-FigureS1-S7/acel70059-sup-0001-FigureS1-S7/acel70059-sup-0001-FigureS1-S7/acel70059-sup-0006-FigureS6.tif]

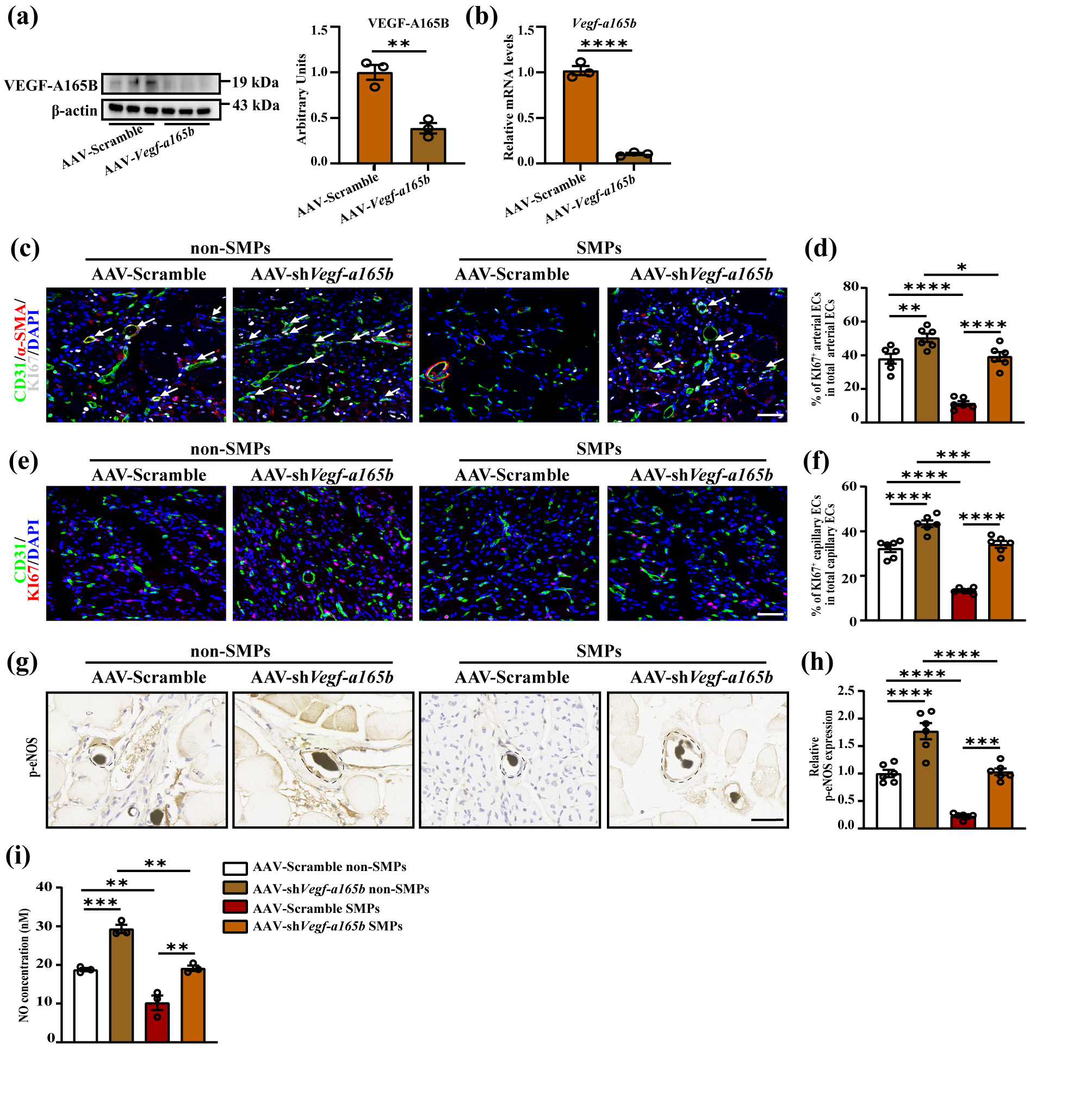

Supplement: Supplementary file 1 — Figure S1. Aging promotes the macrophages senescence in hindlimb skeletal muscle. Gating strategy of macrophages in the hindlimb skeletal muscle. Representative flow cytometry plots for macrophages of hindlimb skeletal muscle stained with CD86, blank control and fluorescence minus one (FMO) control. Representative flow cytometry plots for macrophages of hindlimb skeletal muscle stained with CD206, blank control and FMO control. (d, g) Representative flow cytometry plots (d) and quantification (g) of the percent of SPiDER‐βGal+F4/80+ cells in the hindlimb skeletal muscle (n = 6). (e, h) Representative flow cytometry plots (e) and quantification (h) of the percent of SPiDER‐βGal+CD86+ cells in the hindlimb skeletal muscle (n = 6). (f, i) Representative flow cytometry plots (f) and quantification (i) of the percent of SPiDER‐βGal+CD206+ cells in the hindlimb skeletal muscle (n = 6). Representative flow cytometry plots for macrophages of hindlimb skeletal muscle stained with SPiDER‐βGal, blank control and FMO control. Immunoblot images and quantification for MHCII or LYVE1 protein levels in non‐SMPs and SMPs isolated from hindlimb skeletal muscle of 24‐month‐old mice (n = 3). (l, m) Real‐time PCR analysis of Mhcii (l) and Lyve1 (m) in non‐SMPs and SMPs isolated from hindlimb skeletal muscle of 24‐month‐old mice (n = 3). Unpaired t‐tests. Error bars represent SEM. **, ***, **** and ns denote p < 0.01, p < 0.001, and p < 0.0001, not significant, respectively. Figure S2. Senescent macrophages actively affect proliferation and eNOS phosphorylation of skeletal muscle ECs in vivo. (a, b) Representative CD31 (green), aSMA (red), and KI67 (gray) immunofluorescent images (a) and quantification (b) on gastrocnemius cross sections of mice transferred with SMPs or non‐SMPs at 7 days after HLI (n = 6; scale bar = 50 μm). (c, d) Representative CD31 (green) and KI67 (red) immunofluorescent images (c) and quantification (d) on gastrocnemius cross sections of mice transferred with SMPs [file ACEL-24-e70059-s001.zip › acel70059-sup-0001-FigureS1-S7/acel70059-sup-0001-FigureS1-S7/acel70059-sup-0001-FigureS1-S7/acel70059-sup-0007-FigureS7.tif]
