# Supplementary material for: Senescent Macrophages Promote Age‐Related Revascularization Impairment by Increasing Antiangiogenic VEGF‐A165B Expression
Source: Aging Cell. 2025 Apr 17;24(7):e70059. doi: 10.1111/acel.70059 (PMC12266784; doi:10.1111/acel.70059)
Supplement: Supplementary file 2 — Table S1. [file ACEL-24-e70059-s003.docx]

**Tabel S1.** The details of the mouse primers.

| **Gene** | **Forward** | **Reverse** |
| --- | --- | --- |
| *Rn18s* | GGCCCTGTAATTGGAATGAGTC | CCAAGATCCAACTACGAGCTT |
| *Ccl-8* | CATGGAAGCTGTGGTTTTCCAGA | CCATGTACTCACTGACCCACTTC |
| *Ccl-11* | CATGGAAGCTGTGGTTTTCCAGA | CCATGTACTCACTGACCCACTTC |
| *Cxcl12* | GGCGGTCAAAAAGTTTGCCTT | CAGTTAGCCTTGCCTTTGTTCAG |
| *Cxcl13* | CATAGATCGGATTCAAGTTACGCC | GTAACCATTTGGCACGAGGATTC |
| *Icam-1* | AGTCCGCTGTGCTTTGAGA | CACACTCTCCGGAAACGAATACA |
| *Il-1α* | CGAAGACTACAGTTCTGCCATT | GACGTTTCAGAGGTTCTCAGAG |
| *Mmp10* | GAGCCACTAGCCATCCTGG | CTGAGCAAGATCCATGCTTGG |
| *Tnf-α* | GGTGCCTATGTCTCAGCCTCTT | GCCATAGAACTGATGAGAGGGAG |
| *p15* | GGGCAAGTGGAGACGGTG | GCCCATCATCATGACCTGGATT |
| *p16* | GAAAGAGTTCGGGGCGTTG | GAGAGCCATCTGGAGCAGCAT |
| *p19* | AAGACGGCCTTGCAGGTCA | GCATCTTGGACATTGGGGCT |
| *p21* | AGAAGGTACTTACGGTGTGGT | GAGAGATTTCCCGAATTGCAGT |
| *p27* | TCAAACGTGAGAGTGTCTAACG | CCGGGCCGAAGAGATTTCTG |
| *Cd38* | TCTCTAGGAAAGCCCAGATCG | GTCCACACCAGGAGTGAGC |
| *Sirt1* | GGTTGACTTAGGTCTTGTCTG | CGTCCCTTGTAATGTTTCCC |
| *Sirt2* | AGCAAGGCACCA CTAGCCACC | TGTTCCTCTTTCTCTTTG |
| *Sirt3* | ATCCCGGACTTCAGATCCCC | CAACATGAAAAAGGGCTTGGG |
| *Sirt4* | GTGGAAGAATAAGAATGAGCGGA | GGCACAAATAACCCCGAGG |
| *Sirt5* | CTCCGGGCCGATTCATTTCC | GCGTTCGCAAAACACTTCCG |
| *Sirt6* | ATGTCGGTGAATTATGCAGCA | GCTGGAGGACTGCCACATTA |
| *Sirt7* | AGCATCACCCGTTTGCATGA | GGCAGTACGCTCAGTCACAT |
| *Il-6* | CGTGGAAATGAGAAAAGAGTTGT | GGTAGCATCCATCATTTCTTTGT |
| *Il-1β* | CCACCTTTTGACAGTGATGAGA | GACAGCCCAGGTCAAAGGTT |
| *iNOS* | CACCACCCTCCTCGT TC | CAATCCACA ACTCGCTCC |
| *MCP1* | TTTTTGTCACCAAGCTCAAGAG | TTCTGATCTCATTTGGTTCCGA |
| *Arg1* | CCACAGTCTGGCAGTTGGAAG | GGTTGTCAGGGGAGTGTTGATG |
| *Mgl1* | CAGAATCGCTT AGCCAATGTGG | TCCCAGTCCGTGTCCGAAC |
| *Mgl2* | TTCAAGAATTGGAGGCCACT | CAGACATCGTCATTCCAACG |
| *Mrc1* | CTCTGTTCAGCTATTGGACGC | CGGAATTTCTGGGATTCAGCTTC |
| *Vegf-a* | ACTGGACCCTGGCTTTACTGC | TGATCCGCATGATCTGCATGGTG |
| *Vegf-a165a* | CAGAAAATCACTGTGAGCCTTGTT | CTTGGCTTGTCACATCTGCAA |
| *Vegf-a165b* | CAGAAAATCACTGTGAGCCTTGTT | ATCGGTCTTTCCGGTGAGTCT |
| *Mhcii* | GACACGGTGTGCAGACACAAC | GTCACTGAGCAGACCAGAGTGTT |
| *Lyve1* | GGTGCTGGCTCTCCTCTTCT | GACGTCATCAGCCTTCTCTTCCT |
