# Supplementary material for: Senescent Macrophages Promote Age‐Related Revascularization Impairment by Increasing Antiangiogenic VEGF‐A165B Expression
Source: Aging Cell. 2025 Apr 17;24(7):e70059. doi: 10.1111/acel.70059 (PMC12266784; doi:10.1111/acel.70059)
Supplement: Supplementary file 3 — Table S2. [file ACEL-24-e70059-s002.docx]

**Tabel S2.** Patient characteristics.

| **Patient**  **Numbers** | **Age** | **Sex** | **BMI** | **Diabetes** | **Hyper-tension** | **Coronary**  **artery disease** | **Hyper-**  **lipidemia** | **Ankle Brachial Index** |
| --- | --- | --- | --- | --- | --- | --- | --- | --- |
| 1 | 51 | M | 25.80 | No | Yes | No | No | 1.060 |
| 2 | 53 | M | 23.95 | No | Yes | No | Yes | 1.005 |
| 3 | 54 | M | 24.03 | No | No | No | No | 1.100 |
| 4 | 54 | M | 23.98 | DM II | Yes | No | No | 0.630 |
| 5 | 56 | M | 22.04 | DM II | No | No | No | 0.610 |
| 6 | 57 | M | 21.56 | No | Yes | No | Yes | 0.800 |
| 7 | 58 | F | 26.04 | No | Yes | No | No | 0.800 |
| 8 | 58 | M | 25.71 | DM II | Yes | No | No | 0.665 |
| 9 | 62 | M | 24.91 | DM II | Yes | Yes | Yes | 0.510 |
| 10 | 64 | M | 26.96 | DM II | Yes | Yes | No | 0.920 |
| 11 | 67 | M | 21.45 | DM II | Yes | No | No | 0.385 |
| 12 | 69 | M | 18.18 | DM II | No | Yes | No | 0.795 |
| 13 | 69 | M | 21.48 | DM II | Yes | No | No | 0.725 |
| 14 | 69 | M | 19.53 | No | No | No | No | 0.480 |
| 15 | 69 | M | 19.96 | DM II | No | Yes | No | 0.585 |
| 16 | 69 | M | 21.63 | No | Yes | No | Yes | 0.515 |
| 17 | 70 | M | 23.73 | DM II | No | No | No | 0.885 |
| 18 | 70 | F | 23.50 | DM II | Yes | No | No | 0.780 |
| 19 | 70 | F | 18.73 | DM II | Yes | No | No | 0.510 |
| 20 | 70 | M | 24.03 | DM II | No | No | No | 0.440 |
| 21 | 70 | M | 19.59 | No | Yes | Yes | No | 0.405 |
| 22 | 70 | F | 19.15 | DM II | Yes | No | No | 0.555 |
| 23 | 71 | M | 25.25 | No | Yes | No | No | 0.300 |
| 24 | 71 | M | 23.66 | DM II | Yes | No | No | 0.135 |
| 25 | 74 | M | 22.86 | DM II | Yes | No | No | 0.860 |
