# Supplementary material for: Senescent Macrophages Promote Age‐Related Revascularization Impairment by Increasing Antiangiogenic VEGF‐A165B Expression
Source: Aging Cell. 2025 Apr 17;24(7):e70059. doi: 10.1111/acel.70059 (PMC12266784; doi:10.1111/acel.70059)
Supplement: Supplementary file 4 — Table S3. [file ACEL-24-e70059-s004.docx]

**Table S3.** The details of the overlapping CS-DEGs.

| **Gene ID** | **Gene symbol** | **logFC** | **P.Value** |
| --- | --- | --- | --- |
| 12189 | *Brca1* | -0.55664 | 0.000002 |
| 12534 | *Cdk1* | -0.57791 | 0.000000 |
| 13807 | *Eno2* | -0.57243 | 0.000318 |
| 16832 | *Ldhb* | -0.53087 | 0.032545 |
| 20311 | *Cxcl5* | -0.64634 | 0.011837 |
| 12428 | *Ccna2* | -0.64701 | 0.000000 |
| 16000 | *Igf1* | -1.03797 | 0.000000 |
| 16818 | *Lck* | -0.51588 | 0.015499 |
| 104111 | *Adcy3* | -0.73564 | 0.000000 |
| 17381 | *Mmp12* | -0.51869 | 0.000000 |
| 16009 | *Igfbp3* | 1.01338 | 0.016393 |
| 14254 | *Flt1* | 0.66529 | 0.000367 |
| 13197 | *Gadd45a* | 0.65706 | 0.000000 |
| 11522 | *Adh1* | 1.12401 | 0.012791 |
| 23882 | *Gadd45g* | 0.57212 | 0.000000 |
| 13198 | *Ddit3* | 0.71199 | 0.000000 |
| 11839 | *Areg* | 0.73355 | 0.000000 |
| 16153 | *Il10* | 0.63460 | 0.000000 |
| 20303 | *Ccl4* | 1.13783 | 0.000000 |
| 20306 | *Ccl7* | 0.96323 | 0.000000 |
| 20302 | *Ccl3* | 0.85844 | 0.000000 |
| 26362 | *Axl* | 1.07088 | 0.000000 |
| 14972 | *H2-K1* | 0.87758 | 0.000000 |
| 13653 | *Egr1* | 1.04011 | 0.000000 |
| 17873 | *Gadd45b* | 0.64620 | 0.000000 |
| 16168 | *Il15* | 0.87953 | 0.000000 |
| 12156 | *Bmp2* | 1.53499 | 0.000007 |
| 231991 | *Creb5* | 0.65635 | 0.000000 |
| 20296 | *Ccl2* | 0.81940 | 0.000000 |
| 18035 | *Nfkbia* | 0.64122 | 0.000000 |
